# The scope and extent of literature that maps threats to species globally: a systematic map

**DOI:** 10.1186/s13750-022-00279-7

**Published:** 2022-07-09

**Authors:** Francesca A. Ridley, Emily J. Hickinbotham, Andrew J. Suggitt, Philip J. K. McGowan, Louise Mair

**Affiliations:** 1 School of Natural and Environmental Sciences, Newcastle University, Ridley Building 2, Newcastle upon Tyne, NE1 7RU UK; 2 Department of Geography and Environmental Sciences, Northumbria University, Ellison Place, Newcastle Upon Tyne, NE1 8ST UK

**Keywords:** Biodiversity conservation, Evidence synthesis, Species extinctions, Human footprint

## Abstract

**Background:**

Human activities are driving accelerating rates of species extinctions that continue to threaten nature’s contribution to people. Yet, the full scope of where and how human activities threaten wild species worldwide remains unclear. Furthermore, the large diversity of approaches and terminology surrounding threats and threat mapping presents a barrier to understanding the state of knowledge and uptake into decision-making. Here, we define ‘threats’ as human activities and direct human-initiated processes, specifically where they co-occur with, and impact the survival of, wild species. Our objectives were to systematically consolidate the threat mapping literature, describe the distribution of available evidence, and produce a publicly available and searchable database of articles for easy uptake of evidence into future decision-making.

**Methods:**

Four bibliographic databases, one web-based search engine, and thirteen organisational websites were searched for peer-reviewed and grey-literature published in English 2000–2020. A three-stage screening process (title, abstract, and full-text) and coding was undertaken by two reviewers, with consistency tested on 20% of articles at each stage. Articles were coded according to 22 attributes that captured dimensions of the population, threat, and geographic location studied in addition to methodological attributes. The threats studied were classified according to the IUCN Red List threat classification scheme. A range of graphical formats were used to visualise the distribution of evidence according to these attributes and complement the searchable database of articles.

**Review findings:**

A total of 1069 relevant threat mapping studies were found and included in the systematic map, most conducted at a sub-national or local scale. Evidence was distributed unevenly among taxonomic groups, ecological realms, and geographies. Although articles were found for the full scope of threat categories used, most articles mapped a single threat. The most heavily mapped threats were alien invasive species, aquatic or terrestrial animal exploitation, roads and railways, residential development, and non-timber crop and livestock agriculture. Limitations regarding the English-only search and imperfect ability of the search to identify grey literature could have influenced the findings.

**Conclusions:**

This systematic map represents a catalogue of threat mapping evidence at any spatial scale available for immediate use in threat reduction activities and policy decisions. The distribution of evidence has implications for devising actions to combat the threats specifically targeted in the post-2020 UN Biodiversity Framework, and for identifying other threats that may benefit from representation in global policy. It also highlights key gaps for further research to aid national and local-scale threat reduction. More knowledge would be particularly beneficial in the areas of managing multiple threats, land-based threats to marine systems, and threats to plant species and threats within the freshwater realm.

**Supplementary Information:**

The online version contains supplementary material available at 10.1186/s13750-022-00279-7.

## Background

Species extinctions are occurring at up to 1000 times the background rate [1, 2], and for some taxa these rates are now comparable to previous mass extinction events [3–5]. Unlike the five previous events that were prefaced by large-scale geological and climatic changes, the current pulse of extinction is being driven by human activities [6–9]. Yet, biodiversity contributes vital services to humanity such as climate regulation, food production, and clean water and air provision [10, 11]. Therefore, it is imperative that the human-driven threats to species are reduced to bend the curve of biodiversity loss [12, 13].

Spatial prioritisations are a useful tool for informing a variety of conservation practice and policy interventions. For example, mapping is a highly recommended part of a systematic conservation planning process, and can help to identify where to carry out specific actions and prioritise limited financial and physical resources [14–16]. These maps tend to be local in scope, and coupled with multi-criteria decision making or cost-effective analyses [17, 18]. On the other hand, multi-national and global-scale conservation priority maps have the power to generate public and policy awareness to deliver large amounts of funding to conservation projects in particular areas [16, 19]. For instance, the Critical Ecosystem Partnership Fund awarded US$255 million to conservation projects in Biodiversity Hotspots [20] between 2000 and 2020 [21]. Therefore, conservation priority maps at different scales have differing but equally important functions for conserving biodiversity.

The term ‘threat mapping’ has been used to refer broadly to any spatial representation of the occurrence, intensity, or consequence of threat or threats [16]. This means that the term ‘threat mapping’ has been applied equally to maps of threatened species or extinction risk categories (herein maps of species state, e.g. [19, 20, 22–24]), maps of human-driven activities irrespective of species presence (herein maps of human pressure, e.g. [25–27]), and maps of the spatial co-occurrence between species and threatening human activities (herein threat maps, e.g. [28–30]). Threat mapping for spatial prioritisation has been criticised as being insufficient for making effective conservation decisions [16], yet such critiques have not distinguished between the types of maps described here. Of these three, the latter (threat maps) and their underlying data have the greatest potential to inform threat reduction actions but the availability, characteristics, and utility of which has not yet been discussed or formally reviewed.

A lack of standardised terminology in the literature surrounding threat maps makes the process of finding relevant maps arduous, representing a barrier to understanding the state of knowledge and to uptake in conservation planning and policy decisions. This is not limited to mapping approaches but also pervasive in the definition of threats themselves, for which ‘stress’, ‘impacts’, ‘risk’, ‘drivers’ and ‘footprints’ are often used synonymously [29, 31–33]. Furthermore, many authors refer to processes such as habitat loss and land-use change as a threats [34–37]. Whereas, others consider these processes to be the mechanism by which threatening human activities result in species declines rather than being threats themselves [38, 39]. Rigorous systematic review processes can overcome such variation in language; however, these can be time-consuming, and conservation planning and policy decisions are often made on timescales too short to accommodate their findings [40].

A clarification in terminology is useful here to distinguish maps that: (a) show the spatial coincidence of species and threatening human activities, from (b) other spatial representations of threat. Here, ‘pressures’ are considered to be the human activities themselves that have the potential to become ‘threats’ where they adversely affect wild species. Consequently, threat mapping literature is that which presents the geographic occurrence of threats to species. For example, a study investigating the effect of a human-pressure (x) on a population of species (y) would not be considered a threat mapping study unless it visually presented the geographic distribution of x and y within the study area. In other words, threat mapping research is any investigation that presents the geographic co-occurrence of wild populations of species and the human-driven activities that negatively impact them.

Consolidating and describing the characteristics of the threat mapping literature is a vital next step towards understanding where and how human activities threaten species globally. A diversity of approaches are present in the threat mapping literature, including a range of spatial scales, threats studied, taxonomic groups, and questions asked [29, 32, 41–43]. In addition, the quantity of scientific literature inside and outside conservation science has increased considerably in recent years and continues to do so [44–47]. A simultaneously numerous and fragmented literature contributes to the barrier between research and implementation [44] and presents a high risk of research effort duplication and contradictory findings, as demonstrated in related fields [24, 48]. Moreover, unidentified gaps and clusters in knowledge can result in a distorted understanding of a system [49–51], thus increasing the likelihood that decisions based on such knowledge are flawed. Therefore, given that a major purpose of threat maps is to prioritise limited resources for threat abatement action and awareness, there is an urgent need to consolidate the, yet uncharacterised, threat mapping literature.

There is extensive evidence that research effort in conservation science varies among taxonomic groups [49, 52–61], and some evidence for variation among geographic locations [49, 54–56, 60]. Additionally, it is expected that not all threats will be equally represented within the threat mapping literature, however, to our knowledge, there are no articles that consider differences in conservation research effort among threats. As threat mapping often relies on satellite data, threats that have a remotely observable footprint are expected to be disproportionately prominent in the literature [25, 26, 41]. Whereas, direct exploitative threats are likely to be underrepresented [62, 63], despite biological resource use being the most frequently reported threat in IUCN Red List assessments [64]. Furthermore, despite many spatial articles [65–67], alien invasive species were included in only three cumulative threat assessments found during our protocol development [29, 30, 68]. Therefore, this work has the potential to highlight gaps in knowledge of high benefit to threat abatement efforts.

This systematic map of the literature describes the review process undertaken, the distribution of threat mapping evidence across the world, and the searchable database of threat mapping articles. The systematic map is intended to fill a knowledge gap that has emerged during a wide range of discussions with diverse stakeholders during the development of the Post-2020 Global Biodiversity Framework. Due to the emphasis on reducing the direct threats to species in the post-2020 framework, it is expected that the findings of the map and database of articles will inform these negotiations and the implementation of the framework when it is agreed. The systematic map was produced according to the published protocol [69], with only minor adjustments to the search strategy and eligibility criteria needing to be made, which are described in full and justified below.

## Objectives of the review

The aim of the systematic map was to describe the current distribution of threat mapping literature by collecting and analysing data on the methodological, taxonomic, and geographic extent of articles that have mapped threats to species. Descriptive analyses were used to identify gaps and clusters in knowledge to complement the publicly available database of articles and corresponding meta-data. As the scope of this investigation was existing in-situ threats, any articles published before 2000, articles of historical, future, or potential threats, and theoretical, captive, or lab-based articles were excluded. Furthermore, this analysis specifically considered threat mapping articles, therefore only articles that presented geographic distributions of both the threats and the affected species were included. Examples of how threat maps can be presented are given in Additional file 1.

### Primary question

What is the scope and extent of literature that maps threats to species?

The following are the question elements:

#### Population

The taxonomic scope was any wild animal or plant species globally, in any ecological realm (terrestrial, marine, freshwater). Accepted proxies for the presence of species are detailed in the eligibility criteria.

#### Outcome

The outcome examined was the spatial occurrence of threats. Threats are considered to occur where threatening human activities or direct human-initiated processes co-occur with, and negatively impact, wild species. It is emphasised that the focus of this study was the direct human-driven activities and processes rather than indirect processes or the ecological mechanisms (stressors) that subsequently impact species. For example, articles mapping the occurrence of human-wildlife conflict where the subject of the measured impact was not the wild species (e.g. the impacted subject was human), or articles mapping freshwater quality indicators without specifying a human source of pollution, did not merit inclusion. In contrast, articles mapping retaliatory killing of predators by humans or those mapping agricultural or industrial effluent where species were observed, would both qualify for inclusion.

The IUCN Red List threat classification scheme [38] was used as a guide to categorise the threats studied in each article in a consistent and coherent manner. This allowed threat maps using different methodologies and terminologies to be analysed in the same way, and any threats in the IUCN classification scheme that were unrepresented in the threat mapping literature could be identified. There is evidence to suggest that some of the threats may interact with one another [17, 70–72], which the chosen framework did not capture. However, as the purpose of this investigation was to catalogue the existing evidence, the IUCN threat classification scheme was deemed to be the most appropriate framework to use.

#### Methods

Any primary research that collected and presented data on the geographic occurrence of threats was considered within the scope of this investigation. This included georeferenced presentations of direct or remote observations, spatial modelling results, expert elicitation processes, existing data such as from the IUCN red list or museum archives, or data collected from existing literature. Similarly, all cartographic methods were within the scope. Whereas, schematic representations of the occurrence or gradient of threats without a specific geographic context were outside the scope.

### Secondary questions of the systematic map

Descriptive analyses for the systematic map were structured around the following secondary questions:

1. What is the geographic distribution of the existing literature?
2. What is the taxonomic distribution of the existing literature?
3. Which threats are studied most frequently and how many different threats are considered in each study?
4. How has the extent of knowledge changed over time
5. Where do gaps and clusters in knowledge exist?

## Methods

The following outlines the searching, screening, and data extraction process in the production of the systematic map. This was carried out in accordance with the published protocol [69]. The few changes to the protocol that were made are described and justified below, before describing in full the method that was used in the relevant sub-sections.

### Deviations from the protocol

1. An alternative strategy was adopted to search some organisational websites. Searches conducted via (internal) website search boxes often generated materials irrelevant to this protocol, such as press releases and educational resources. Some websites also had additional filtering features to aid search efficiency and a publications page on the website, separate to the search function. Therefore, where the proposed search was found to be inefficient and other such tools were available, an alternative strategy was used. The specifics of how each organisational website was searched and screened were recorded (Additional file 2).
2. The criteria for including articles at title level was expanded as many potentially relevant articles would have been excluded under the previous guidance. In the protocol it was stated that ‘if there was insufficient information present in the title to conclusively exclude a study, all articles pertaining to an effect of human-driven threats on species, or prioritising conservation efforts will be screened again at abstract level’. Yet at the title screening stage, this guidance was found to be too restrictive. For example, titles that described the spread of human-driven pressure without referring to species, or those titled as conservation status assessments would have been excluded at this stage under the previous guidance. Therefore, expanding the scope of titles included at this stage was deemed necessary. The updated guidance related to inclusion of articles at title level is described fully in ‘Article screening and eligibility criteria’.
3. Exclusion criteria 2B was changed from “Excluded if ecological stress is measured without being considered a proxy for a particular human activity” to “Excluded if the threat studied is relevant and studied in-situ but the occurrence was not mapped onto a geographic distribution”. When applying the criteria it was found that the original criteria 2B was already represented under criteria 2A, whereby articles were excluded due to an absence of data on a relevant threat. Meanwhile, articles that undertook an otherwise relevant piece of research but did not present the findings in a spatially explicit way were abundant. Therefore, the new structure to the eligibility criteria was considered to better represent the reasons why articles were excluded.
4. The protocol included one subsequent round of snowballing within the search strategy. Snowballing was piloted on a sample of 574 articles (54% of the total relevant articles), in which all the literature cited in this sample was collected and any articles retrieved in the original search were removed. This process yielded 16,850 novel documents. Assuming that the three-stage screening would be completed at the same rate for the snowballed articles as the main search, the time required to screen and extract meta-data from the snowballed articles was estimated to be 58 weeks. Therefore, completion of snowballing would likely result in the findings from the main search being outdated at the time of publication and the commitment to publish within 2 years of search commencement being breached. Consequently, the decision was taken not to complete the snowballing. Nevertheless, we acknowledge the potential of this technique for finding other relevant literature and encourage interested researchers with the required knowledge to do so.

### Search for articles

Databases of commercially published and grey literature were searched in accordance with the published protocol. Two candidate searches were identified that retrieved all articles in a test-list of benchmark articles, and were compared using a title-level screen. The final search was chosen as a balance of search sensitivity and specificity. For full details of the scoping searches that were undertaken in search string development, tests of search comprehensiveness, and how the search string was adapted for each database see the published protocol [69].

#### Search string

The final search string was as follows. This exact string was used to search SCOPUS and details of how it was adjusted to suit other databases can be found in Additional file 2.

Search String: (pressure OR threat OR risk OR stress OR footprint) *AND* (species OR ecosystem OR wildlife OR fauna OR flora OR {spp}. OR {sp.}) *AND* (hotspot* OR map* OR geographic* OR “gis” OR “spatial distribution” OR “spatial overlap” OR “spatial separation” OR “spatial dynamics” OR “spatial variation” OR “spatial framework” OR “spatially explicit” OR geospatial) *AND* (conservation OR biodiversity).

#### Search limitations

The final search was used to search publication databases, search engines and grey-literature repositories in English for articles published between 2000 and 2020 inclusive. Inclusion of non-English language articles was not considered feasible here due to translation resource restrictions. Furthermore, carrying out snowballing could have retrieved many more relevant articles. Therefore, we openly encourage interested researchers with the necessary skills to repeat our protocol for non-english languages and snowballing searches.

Through further consideration of the eligibility criteria, it was determined that two articles in the original test set of articles did not merit inclusion due to nuances in the definitions of future threats, and mapping [73, 74]. As these nuances were not detectible in the title, abstract or keywords of the papers, this is not considered to have compromised the comprehensiveness of the search. In addition, having ineligible articles in the test-set may only have resulted in a higher number of irrelevant articles being found rather than limiting the number of relevant articles found. Therefore, the overall impact of this on the comprehensiveness of the systematic map is considered to be minimal.

#### Publication databases

SCOPUS, ProQuest natural Science Collection, and Web of Science Core Collection were searched for published peer-reviewed articles by title, abstract and keywords using the subscriptions of Newcastle University. For specifics of the citation indexes used and how the search terms were adapted for each database please see ‘Additional file 2’ or the published protocol [69].

#### Search engine

Google Scholar was used to identify grey literature by searching the titles with the simplified search string of: (pressure OR threat OR footprint) AND (species OR ecosystem OR wildlife). A title-level search has previously been found more effective than searching the full-text on Google Scholar [75]. The results were ordered by relevance and the first 500 gathered.

#### Grey literature searches

ProQuest Natural Science collection was specifically searched for non-commercially published dissertations and theses, government and official publications, reports, and working papers using the same search string as the ProQuest Natural Science commercially published literature search.

#### Website searches

The following organisational websites were searched for additional grey literature. A bespoke approach was taken that utilised additional search features and repositories within each website. For full details of how each organisational website was searched see Additional file 2.

- World Wildlife Fund for Nature (WWF) [76]
- United Nations Environment Programme World Conservation Monitoring Centre (UNEP-WCMC) [77]
- Convention on Biological Diversity (CBD) [78]
- International Union for the Conservation of Nature (IUCN) [79]
- Intergovernmental Panel on Biodiversity and Ecosystem Services (IPBES) [80]
- Royal Society for the Protection of Birds (RSPB) [81]
- Fauna and Flora International (FFI) [82]
- The Nature Conservancy [83]
- Conservation International [84]
- Birdlife International [85]
- Blue Ventures [86]
- The Audubon Society [87]
- Society of Conservation Biology (SCB) [88].

#### Search results

EPPI-Reviewer-Web [89] was used to assimilate and de-duplicate the search results as well as coordinate the screening stage among multiple reviewers. EPPI-Reviewer-Web identifies duplicates based on a similarity algorithm. The threshold for automatic removal of duplicates was set at 0.9, whereby any articles with a similarity score greater than 0.9 were automatically removed. Articles that the duplicate removal tool identified as potential duplicates but had a similarity score of less than 0.9 were checked manually. The application creators advise that duplicates are unlikely to be incorrectly identified above a similarity threshold of 0.8. Therefore, we are confident that no novel articles were incorrectly excluded as duplicates.

To gather the full-text documents of all articles included at the full-text screening stage a combined strategy was used, utilising the Endnote full-text finding tool and manual searching.

### Article screening and eligibility criteria

#### Screening process

A three-stage screening process (Title, Abstract, and Full-text) was undertaken using EPPI-reviewer-web [89]. At each stage, the articles were compared against the eligibility criteria and a decision made about whether the article was relevant. The following guidance was provided to reviewers about how to manage missing information at each stage.

If there was insufficient information to conclusively exclude a study at title level, articles were included if a potentially relevant human-driven pressure and potentially relevant population were present or alluded to in the title. Terms such as species, ecosystem, and population were considered to sufficiently allude to species and a simultaneous reference to conservation or management was considered to sufficiently allude to a threat. Alternatively, titles that described a relevant human-driven pressure were considered to sufficiently allude to threats, where information on the subject of the pressure was either absent or was not obviously human. Due to the wide variety of titles expected to be retrieved, reviewers were also advised that if they strongly suspected that the study contained the relevant information despite not fitting either of those descriptions, then the study should be reviewed again at abstract level.

At the abstract screening stage, reviewers were advised that where insufficient information was present to confidently exclude a study it should be screened again at full-text level. However, abstracts that were entirely narrative and did not contain any suggestions of primary research, were concluded to be narrative reviews and excluded based on study type.

#### Consistency checking

Two reviewers (FAR and EH) were used to carry out the screening process to test the consistency of study classification. At each screening stage (Title, Abstract, and Full-text) a random 20% of articles were allocated to a second reviewer for double screening via EPPI-reviewer-web [89]. Cohen’s kappa [90] was used to compare the proportional agreement between the two reviewers based on two possible coding outcomes: ‘Include’ and ‘Exclude’. At title and abstract level, FAR re-examined all disagreements and, where the reason for disagreement was not obvious (e.g. a clear mistake by either reviewer), the study was included to the subsequent screening stage. At full-text level all disagreements were reconciled by a discussion between the two reviewers. At no point during the screening process were the reviewers involved in decisions regarding the inclusion of their own authored work.

There was weak to moderate agreement between reviewers at the title, abstract, and full-screening stages (Table 1, [91]). A k value of 0.41 is considered moderate by some authors [90, 92], meanwhile others have found k to be limited by low numbers of potential coding outcomes and observer accuracy [93]. In particular, it was found that with two coding outcomes the maximum k was 0.8 and could only be achieved with an observer accuracy of 95% [93]. Therefore, given the wide contextual scope of relevant articles and the level of uncertainty associated with the title screening stage, a k greater than 0.5 was deemed acceptable for the title-level screen.

**Table 1:** Results from consistency testing at each stage of the screening process. N indicates the number of articles compared at each stage (20% of the total screened at each stage). K is the measure of inter-rater reliability (Cohen’s kappa [90])

	n	Exclusion rate (%)		Agreement (%)	k
		Reviewer 1	Reviewer 2		
Title	2804	54	59	76	0.51
Abstract	1368	62	73	84	0.64
Full-text	626	66	64	85	0.66

#### Eligibility criteria

Decisions on whether articles were included or excluded at each stage were made based on the following criteria. For examples of articles that challenged the exclusion criteria and how they were dealt with see Additional file 3.

1. *Eligible population*
  The taxonomic scope of this study was any wild species of animal or plant globally, in any country or ecological realm (terrestrial, marine, freshwater). Evidence of the presence of individuals or groups of such species was an essential criteria for study inclusion. This included direct observations, remotely sensed observations, modelled distributions, and expert-derived species range maps. Pre-defined priority areas for biodiversity conservation, such as a Biodiversity Hotspot or some protected areas were considered to be valid proxies for species presence. A pre-defined priority area was defined as an area of conservation importance identified in previous work by any author, due to the presence, richness or density of animal or plant species. Examples of acceptable proxies for population presence did not deviate from the protocol and are reiterated in Additional file 3. Modelled species distributions were only considered sufficient evidence of species presence if data on species presence within the study site were included in the model input. For example, predictions made using solely environmental analogues were deemed insufficient.
  *Criteria 1A* Excluded if no evidence was given for the presence or distribution of a relevant species within the study area.
  *Criteria 1B* Excluded if the evidence provided was predictively modelled without evidence for species presence at the study site e.g. habitat suitability for the purpose of reintroduction.
2. *Eligible outcome*
  The spatial occurrence of threats to species. As defined above, threats occur where species are exposed to threatening human activities and human-initiated processes. Where articles included multiple ‘threats’ at least one needed to fit the definition used here to qualify for inclusion. If the threat studied was listed on the IUCN classification scheme but was not a result of human action (e.g. geological events) it was excluded. Articles on threats such as fire, extreme weather and disease were only included if they were specifically human-induced within the context of the study. For example, articles of extreme weather were included if they were studied as a consequence of climate-change.
  *Criteria 2A* Excluded if no data on where species and human-driven pressures co-occur were presented or the ‘threat’ considered did not fit the definition used here.
  *Criteria 2B* Excluded if the threat studied was relevant and studied in-situ but the occurrence was not mapped onto a geographic distribution.
  *Criteria 2C* Excluded if the threat considered was not human-driven.
3. *Eligible study type*
  Only primary research published 2000–2020 inclusive in English was included, which may use either primary data, or pre-existing datasets. Exceptions could be made in the case of systematic reviews and meta-analyses where data from primary work was collated and re-analysed. Only articles that assessed current threat distributions were included, as opposed to projected future, potential, or historical distributions. Otherwise, all data collection and cartographic methods were included.
  *Criteria 3A* Excluded if the article was a narrative review in which no new synthesis of data was presented.
  *Criteria 3B* Excluded if the presented distribution represented a historical, future, or potential distribution of threat to species.
  *Criteria 3C* Excluded if the spatial context could not be determined (e.g. due to insufficient reporting or schematic presentation), or the threat was not studied in-situ (e.g. theoretical, lab-based, or experimentally applied).

### Study validity assessment

The validity of individual articles was not assessed beyond study eligibility based on the above criteria, which were written based on the ability of the article to show where species and threatening human activities co-occur geographically. Nevertheless, information on study design and data type was collected for each study allowing for future critical appraisal of the methodology.

### Data coding strategy

Coding of the articles included at the full-text stage was performed by completing the pre-designed data collection tool which formed the basis of the database, where each study occupied a single row (Additional file 4). Meta-data were extracted in terms of bibliographic information, study characteristics (study design, ecological realm, spatial scale, spatial resolution, geographic location), threat characteristics (data type, method of collection or synthesis, data source, threats mapped, number of threats mapped, the thematic precision of threats mapped), and population characteristics (data type, method of collection or synthesis, data source, taxonomic resolution, taxonomic scope and taxonomic group). Data were only collected from the main text and supplementary material of each article; there was no subsequent follow up with authors to clarify missing information. A topology with full lists of potential outcomes and examples for each variable coded is provided in the coding tool (Additional file 4).

#### Coding threats

Threats were classified according to the IUCN Red List threat classification scheme [38] at the second level of thematic precision in the framework hierarchy, where level 1 was the lowest level in precision and level 3 was the highest. For example, bushmeat harvesting was classed as ‘Biological Resource Use’ at the first level of precision (level 1) and ‘Hunting and Collecting Terrestrial Animals’ at level 2. Therefore, for a study that mapped the locations of observed hunting activity, the ‘Threat’ would be coded as ‘Hunting and Collecting Terrestrial Animals’ and the ‘Threat precision’ would be coded as ‘Level 2’.

#### Other and unspecified threats

The threat classification scheme was adapted to acknowledge where threats were studied at a precision lower than level 2 or were not captured by the IUCN criteria despite being relevant according to the criteria above. If threats were mapped at level 1 precision without further clarification as to what activities this included, the threat was considered ‘unspecified’. For example, if a study mapped agricultural land-use without specifying if the production was timber, non-timber, or livestock, the threat was classed as ‘Other or unspecified agriculture and aquaculture’ and the threat precision classed as ‘level 1’. If the threat satisfied the definition of threats used here but wasn’t covered by the IUCN classification, the category ‘other’ was applied. For example, types of fencing that were not otherwise defined under the IUCN threat classification scheme were classed as ‘Other or unspecified linear infrastructure’.

#### Coding consistency

The coding was completed by two reviewers (FAR and EH) to ensure consistency. All included articles were coded by the primary reviewer, and 20% by a second reviewer. Each reviewer independently reviewed all disagreements on coding before the remaining disagreements were discussed and resolved collaboratively. Any necessary clarifications were added to the coding tool.

### Data mapping method

#### Searchable database

All included articles, coded meta-data and bibliographic information have been made available as an excel workbook (Additional file 4) and as an online interactive choropleth map https://naturalandenvironmentalscience.shinyapps.io/ThreatMapping_SM/. The interactive choropleth map was constructed using the leaflet [94] and shiny [95] packages in R (version 4.1.1, [96]). The online interactive map allows users to filter the dataset by any of the meta-data, view the number of articles per country or marine territory, and download a list of citations for their selection.

#### Visual mapping of the meta-data

Summary figures and tables were produced to complement the searchable database and visually map the quantity and quality of evidence relevant to the primary and secondary questions. The taxonomic distribution was visualised using Sankey diagrams that highlighted the relationships among taxonomic group, taxonomic resolution, and taxonomic scope for both animal and plant kingdoms. Co-occurrence matrices were used to identify gaps and clusters in research effort, and observe the linkages between spatial distribution, taxonomy and threats.

To present the geographic distribution of evidence, the geographic location of the study area was collected in data coding. The geographic location was coded either as the country boundary [97] for terrestrial and freshwater articles or as the marine territory [98] for marine articles. Where study areas spanned more than one ecological realm, a judgement was made as to which was the most relevant for coding the geographic location.

## Review findings

### Review of descriptive statistics

#### Searching and screening

**Fig. 1:**
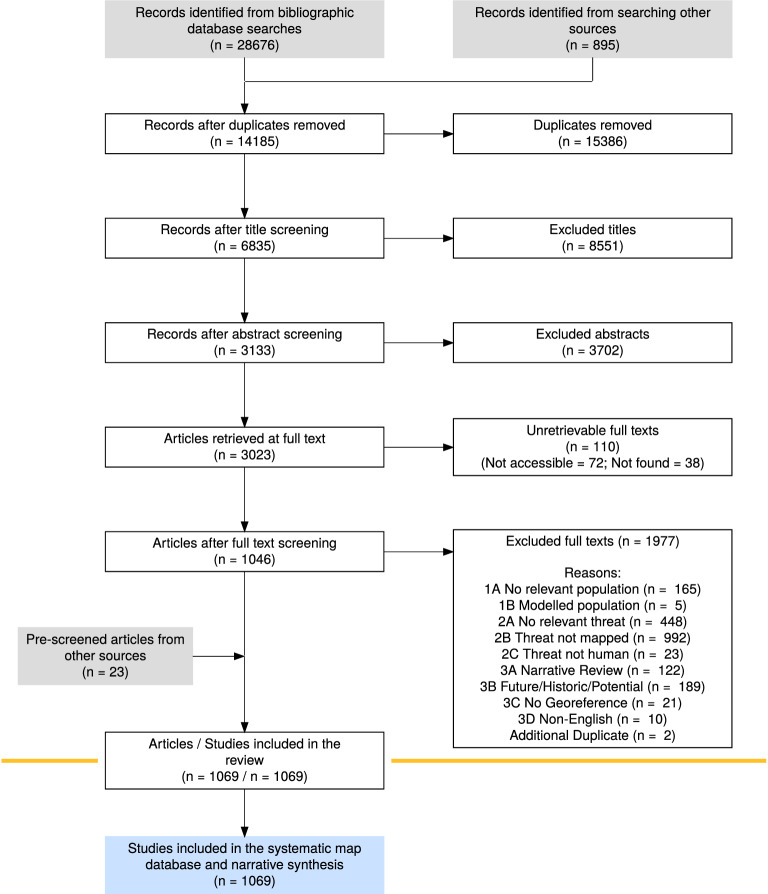
The flow of articles through the screening process generated in accordance with the ROSES Reporting standards for systematic evidence synthesis [99]. As all included articles had to present the findings of primary research, all included articles were scientific studies. ‘Records identified from searching other sources’ refers to articles found in grey literature searching via Google Scholar and ProQuest. ‘Pre-screened articles from other sources’ indicates the articles found through searching organisational websites

The six peer-reviewed and grey literature databases collectively yielded 29,572 articles. Of these, 15,386 were duplicates and removed, leaving 14,185 articles to be screened (Fig. 1). In screening, 6835 (48%) were deemed potentially relevant at title-level, 3133 (46%) at abstract-level, and 1046 (33%) at full-text level (Fig. 1). 110 full texts were irretrievable whereby the full-text of the article could not be found, or it was inaccessible publicly or via the subscriptions used. Of the 1977 excluded at full-text level, 22 were originally included at the full-text screening stage but in light of additional information found at the coding stage both reviewers agreed that these articles did not satisfy the eligibility criteria. Combining the 23 articles found through searches of organisational websites resulted in 1,069 threat mapping articles to be included in the final systematic map (Fig. 1).

Rates of article relevance across the searched sources was low (1.9%–11.3%, Table 2). The source that contributed the highest number of articles to the final systematic map was SCOPUS (881/1069). However, high rates of duplication were recorded for Web of Science Core Collection (WOS) and the search of ProQuest Natural Science Collection for peer-reviewed published articles (ProQuest_Pub_), making the independent ability of WOS and ProQuest_Pub_ to retrieve relevant threat mapping articles uncertain. The search of ProQuest Natural Science Collection for grey literature (ProQuest_Grey_) had the highest rate of relevance in terms of the number of de-duplicated results that were included in the systematic map (Table 2).

**Table 2:** The results of literature searching and duplicate removal, indicating the number of articles from each source that were included after the full-text screening stage

Source	Date Searched	Total results	Duplicates removed	Relevant full-texts	Relevance rate (%)
SCOPUS	15.9.20	10,646	40	881	8.3
Web of Science Core Collection	15.9.20	8953	6544	137	5.7
ProQuest Natural Science (Published)	15.9.20	9078	8690	11	2.8
ProQuest Natural Science (Grey Literature)	15.9.20	395	40	26	11.3
Google Scholar	15.9.20	500	72	13	3.0
Organisational Websites	19.4.21–27.4.21	1238	0	23	1.9

Relevance is the percentage of de-duplicated results that were included after full-text screening. An additional 22 articles were excluded during the coding stage

At full-text stage most articles were excluded on the outcome component of the eligibility criteria. 986 articles (50% of those excluded at full-text level) investigated the occurrence of threats but did not map the findings. Meanwhile, for 444 articles (23%) the threat studied was found not to meet the definition used here (Fig. 1). Moderate numbers were excluded due to the lack of a relevant population (159), the study not being a primary synthesis of data (119), or because the study investigated historical, future, or potential threats (186, Fig. 1). For a full list of articles excluded at full-text level and the criteria that they were excluded on see Additional file 5.

#### Institutions and article types

The articles included in the final systematic map included 1011 journal articles, 22 reports, 15 theses, 13 conference proceedings, six dissertations, one book section, and one generic resource. The most common publications were PLOSOne and Biological Conservation (87 and 80 articles respectively), while the remaining 902 articles were split between 302 publications (Table 3).

**Table 3:** The distribution of articles among publications

Publication name	Number of articles
PLOSONE	87
Biological Conservation	80
Science Of The Total Environment	33
Diversity And Distributions	31
Conservation Biology	29
Biodiversity And Conservation	22
Global Change Biology	22
Journal Of Applied Ecology	20
Ecological Applications	17
Ecological Indicators	17
Ocean And Coastal Management	17
Ecosphere	14
Environmental Management	13
Journal For Nature Conservation	13
Oryx	13
Endangered Species Research	12
Journal Of Environmental Management	12
Environmental Monitoring And Assessment	11
Biological Invasions	10
Proceedings Of The National Academy Of Sciences Of The United States Of America	10
Sustainability(Switzerland)	10
Other	576

The articles were primarily observational (1043 articles) with few being systematic reviews (16 articles) or experimental (10 articles). Experimental articles were defined here as any investigation that manipulated variables regardless of tight controls, such as threat exclusion experiments. Qualitative metrics were more commonly used than quantitative metrics to map both the outcome (threats) and the population (600 versus 469 for threat metrics and 754 versus 315 for population metrics).

### Temporal distribution

**Fig. 2:**
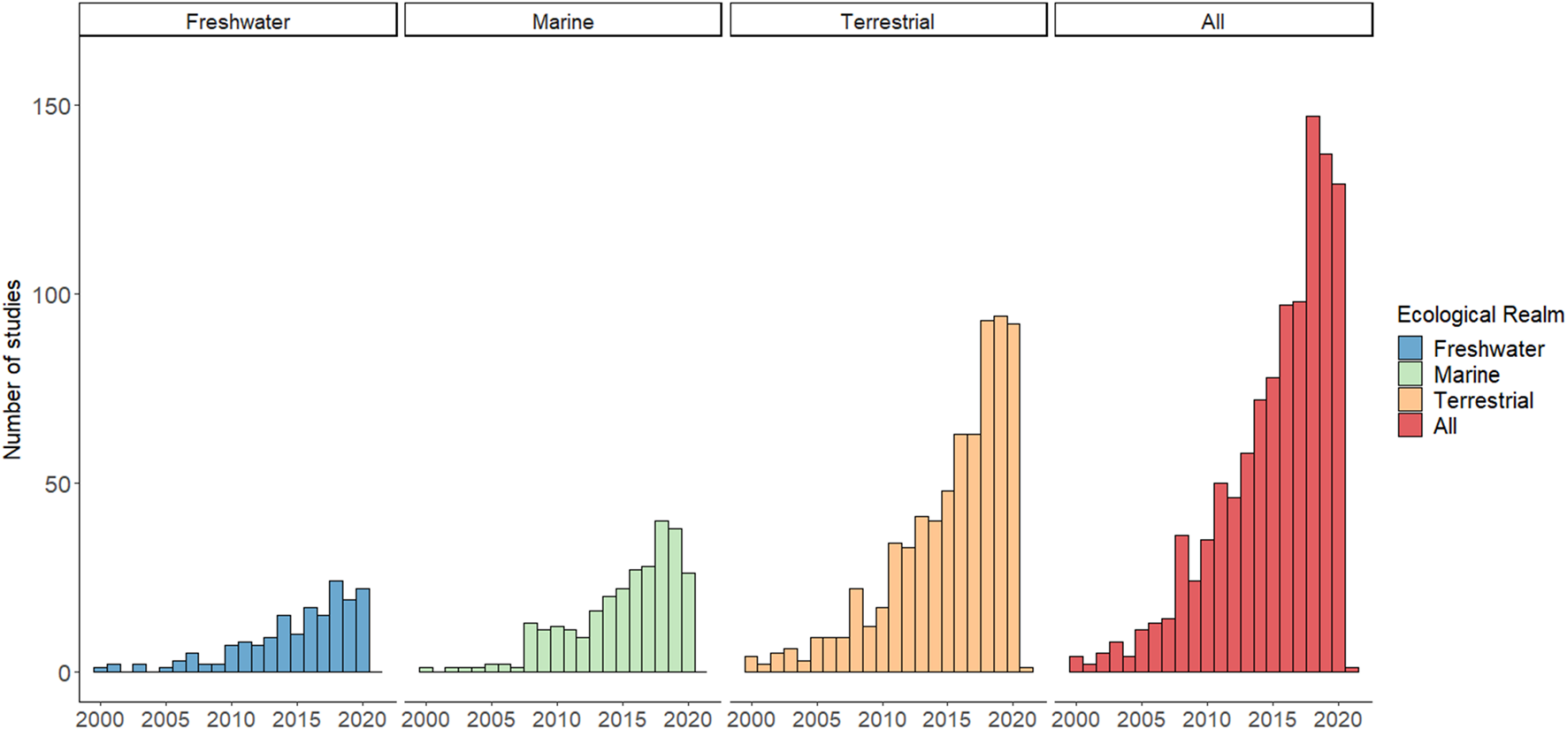
The number of threat mapping articles published in each year by ecological realm. Some articles were relevant to more than one ecological realm so the total published in each year is less than the sum of the number published on each ecoregion for that year. Due to the final date of literature searching being 15.9.2020, the data for both 2020 and 2021 were incomplete

Of the literature retrieved, the number of threat mapping articles published annually has increased over the last 20 years. Five articles were published per annum across 2000–2004. From there, the number of published articles increased annually by 39% on average, to a maximum of 149 articles in 2018 (Fig. 2). As the main search was completed on the 15th September 2020, both 2020 and 2021 are incomplete.

#### Representation of the major ecological realms

The quantity of threat mapping articles was not distributed evenly among the three major ecological realms. Articles relevant to the terrestrial realm outweighed those studying marine or freshwater environments (700, 282, and 171 respectively). Where articles investigated threats to a system in the boundary between these realms (e.g. estuarine or mangrove species) or covered a wide landscape including multiple of these realms, the codes for all relevant ecological realms were applied. Of these boundary-spanning articles, 47 occupied freshwater and terrestrial zones, 18 marine and terrestrial, 5 freshwater and marine, and 7 were relevant to all three.

#### Distribution among spatial scales

The number of articles generally decreased with increasing spatial scale, with the exception of global-scale articles that were conducted at a similar frequency to national and multi-national scale articles. Overall, 345 articles (32%) were conducted at a local scale (< 10,000 km^2^), 333 (31%) at a sub-national scale (> 10,000 km^2^ within a single country), 123 (12%) at a national scale (an entire country extent, irrespective of area), 124 (12%) at a multi-national scale (> 10,000 km^2^ across multiple countries), 44 (4%) at a continental scale and 100 (9%) at a global scale. Consequently, 75% of all threat mapping articles found were conducted at a national scale or below.

**Fig. 3:**
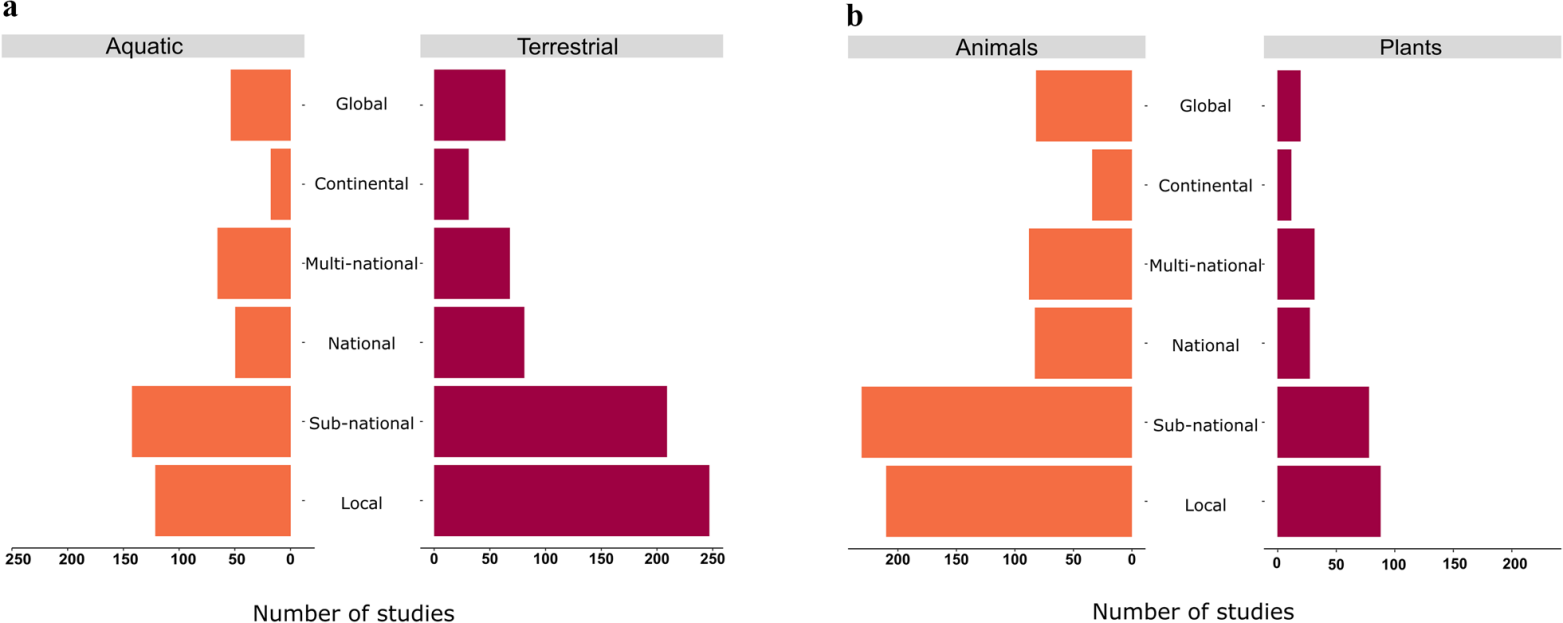
The distribution of evidence among spatial scales, compared for expected sources of bias **a** Ecological realm, **b** Taxonomic Kingdom. Some articles covered more than one ecological realm and studied species from more than one Kingdom

The distribution of articles across spatial scales followed a similar pattern across the compared sub-groups (aquatic versus terrestrial realms and animal versus plant species, Fig. 3). Almost twice as many articles were conducted on the terrestrial realm as the aquatic realms (marine and freshwater), yet the proportion performed at each spatial scale was approximately similar. Likewise, there were almost three times as many articles on animals as plants, yet the proportions of evidence at each spatial scale were almost identical (Fig. 3).

### Mapping the quantity of articles relevant to the question

#### The geographic distribution of threat mapping literature

Based on the literature retrieved, threat mapping research effort was heterogeneously distributed worldwide. For terrestrial and freshwater threat maps it was found that Asia was represented in the most articles (187) followed by North America (156), Europe (150), Africa (122), South America (110), and Australasia (30). Meanwhile in marine applications, the North Atlantic was represented in 70 articles, the Mediterranean Sea in 48, the North Pacific Ocean in 39, the Arctic Ocean in 9, and the Caspian Sea in 2. While, the South Pacific Ocean (31), Indian Ocean (26), South Atlantic Ocean (14), and South China Sea (8) occurred in 79 articles collectively. Antarctica and the Southern Ocean occurred in 3 marine articles and 1 terrestrial or freshwater study.

**Fig. 4:**
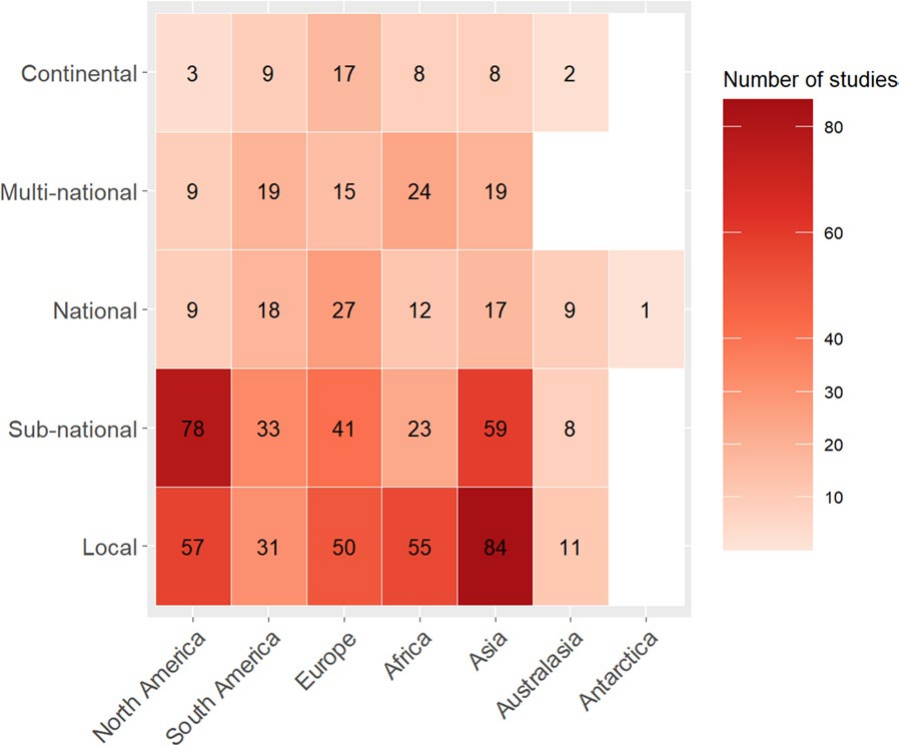
The distribution of terrestrial and freshwater articles among continents and spatial scales

The relative proportions of terrestrial and freshwater articles conducted at each spatial scale differed among geographic regions. The preference for local or sub-national-scale threat maps in these two realms was most pronounced for Asia and North America (76% and 87% respectively) and remained present for Europe, Africa, and South America to a lesser extent (61%, 64% and 58% respectively Fig. 4). Articles of the African continent were more often mapped on a multi-national scale than a sub-national scale (Fig. 4). Furthermore, of all articles conducted on a national scale, 29% occurred in Europe (Fig. 4).

**Fig. 5:**
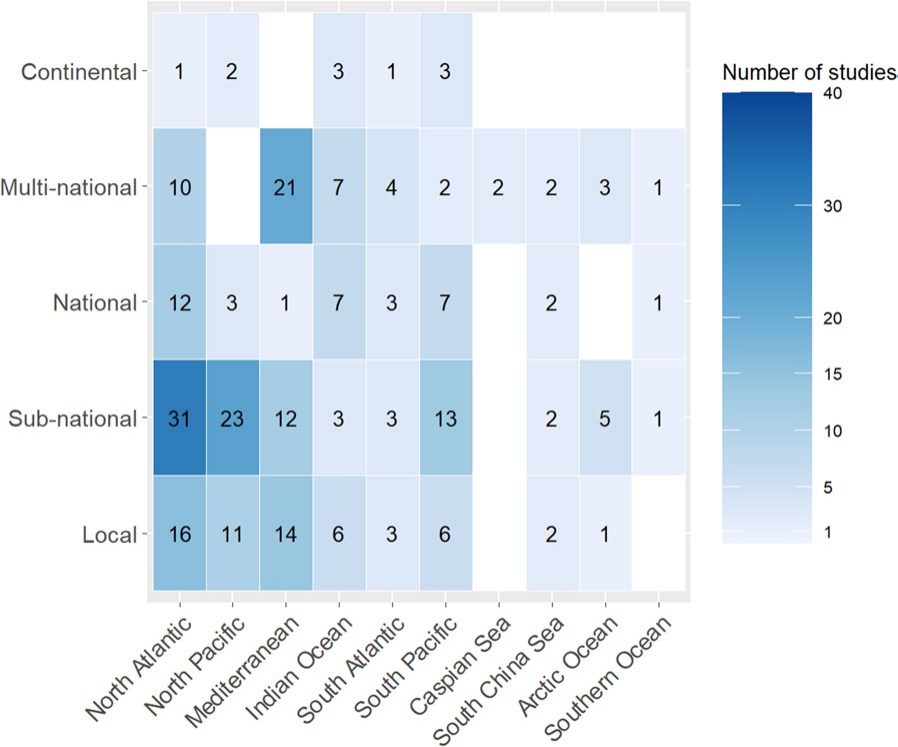
The distribution of marine articles among geographic regions and spatial scales

Threat maps on marine regions were more evenly distributed among spatial scales than terrestrial and freshwater threat maps. Sub-national scale articles formed the highest proportion of those conducted in the North Atlantic (44%), North Pacific (59%), and South Pacific Oceans (42%, Fig. 5). Meanwhile threats to species in the Mediterranean Sea were more likely to be multi-national (44%, Fig. 5). Substantive differences among spatial scales were not observed for other marine regions, although overall numbers of articles for these regions were low, making meaningful comparisons difficult.

**Fig. 6:**
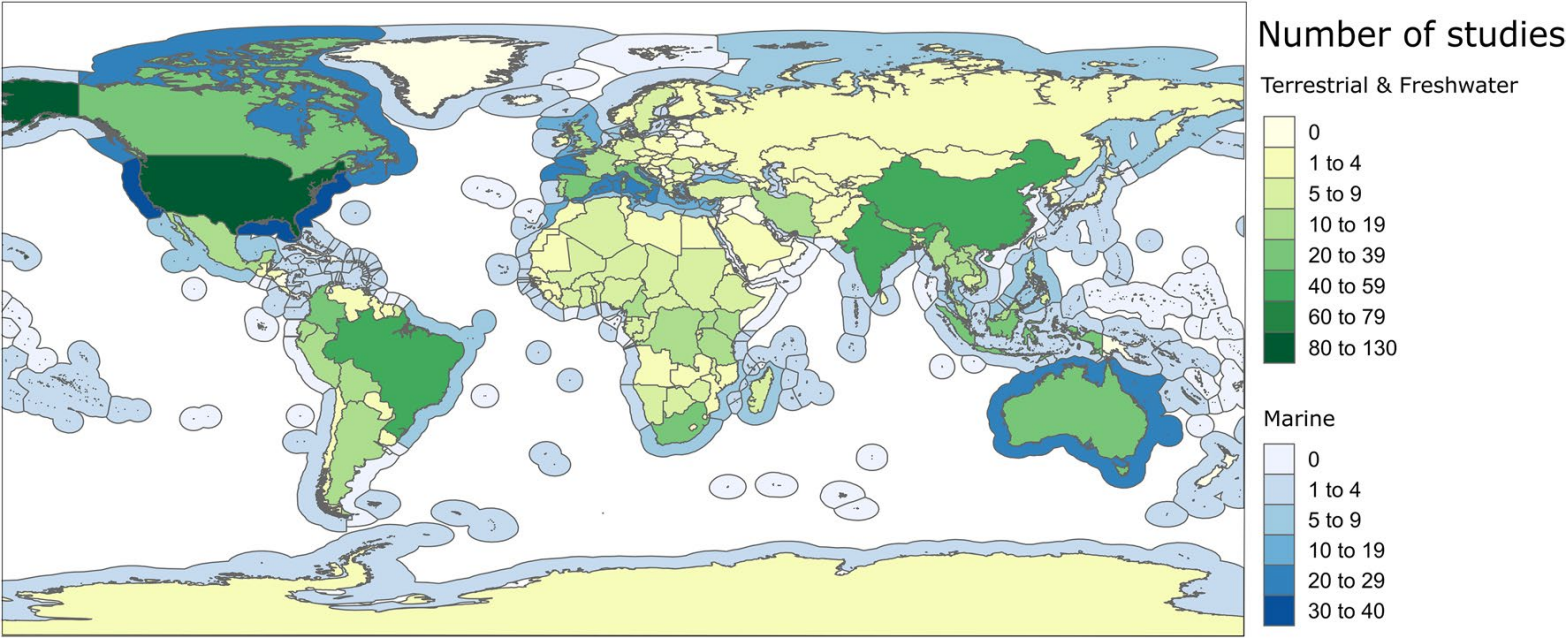
The geographic distribution of the 925 threat mapping articles conducted at a multi-national scale or below

Threat mapping evidence was found covering 144 countries and 160 marine territories. However, the number of articles per country tended to be low; 50% of countries had five or fewer articles and 107 were absent of evidence. The United States of America was found to be the most heavily studied country (124 articles) and marine territory (36 articles, Fig. 6). The next most frequently studied countries in the terrestrial or freshwater realms included Brazil (52 articles), China (51 articles), India (48 articles), and Spain (36 articles, Fig. 6). Therefore, research tended to be clustered around western countries, with some exceptions in large, rapidly-developing countries.

The average number of articles per marine territory was also low, with 50% of marine territories occurring in 2 articles or fewer and 91 marine territories being absent of evidence. The most heavily studied marine territories were the USA (36), Italy (28), Spain (23), Canada (21), Australia (20), and France (20), with the UK and many other Mediterranean marine areas (Greece, Tunisia, Croatia, Morocco, Cyprus, Turkey, Albania, Bosnia and Herzegovina and Slovenia each represented in 10 to 20 articles (Fig. 6). For a full table of the countries and marine territories mapped and the number of articles representing each, see Additional file 6.

#### Taxonomic distribution

##### Distribution between Kingdoms

Most applications of threat mapping focused on animal species alone (664 articles) with fewer that focused on threats to plant species (193 articles) and 65 articles presented data on both animal and plant species. 147 articles used a valid proxy for species presence, in which 97 mapped threats within a protected area and 57 mapped threats within other high biodiversity areas (Table 4).

**Table 4:** The number of articles mapping threats to each taxonomic class

Kingdom	Class	Number of
Animal	Mammal	287
	Bird	171
	Fish	144
	Invertebrate	111
	Reptile	86
	Amphibian	70
	Other	29
	Total	729
Plant	Magnoliopsida	37
	Liliopsida	27
	Pinopsida	9
	Bryopsida	2
	Polypodiopsida	2
	Other	158
	Total	258
Other	Pre-calculated high biodiversity area (PCHBA)	57 (77)
	Protected Areas	97 (160)
	Total	147

Some articles mapped multiple species from different classes and kingdoms, making the total number of articles for each kingdom different to the sum of the articles for each class. The number of articles that mapped a conservation priority area have been denoted as: the number of articles in which the pre-calculated priority area was used as a proxy for population presence (outside parentheses) followed by the total number of papers in which the priority area was mapped, (i.e. including those where other evidence for population presence were also used, inside parentheses)

##### Distribution within Kingdoms

Within the animal kingdom, mammals occupied the highest number of articles (287) followed by birds (171), fish (144) and invertebrates (111), while reptiles and amphibians occupied 86 and 70 articles respectively (Table 4). 29 articles that mapped threats to animals either grouped species by non-taxonomic characteristics (e.g. extinction risk) or did not specify the taxonomy of the included animal species (Table 4).

Of the 258 articles that mapped threats to plants, 181 mapped threats to vascular plants (including forest). 87 articles mapped broad habitat types or unspecified vegetation, 2 articles mapped threats to bryophytes, and 21 mapped threats to other plant species, whereby taxonomy wasn’t specified or species were grouped by non-taxonomic characteristics. Furthermore, among vascular plants, forest (77 articles), flowering plants (36), grasses (27), unspecified tracheophytes (27), and mangroves (22) occupied considerably more threat mapping articles than conifers (9) or ferns (2 articles, Table 4).

##### Taxonomic scope and resolution

**Fig. 7:**
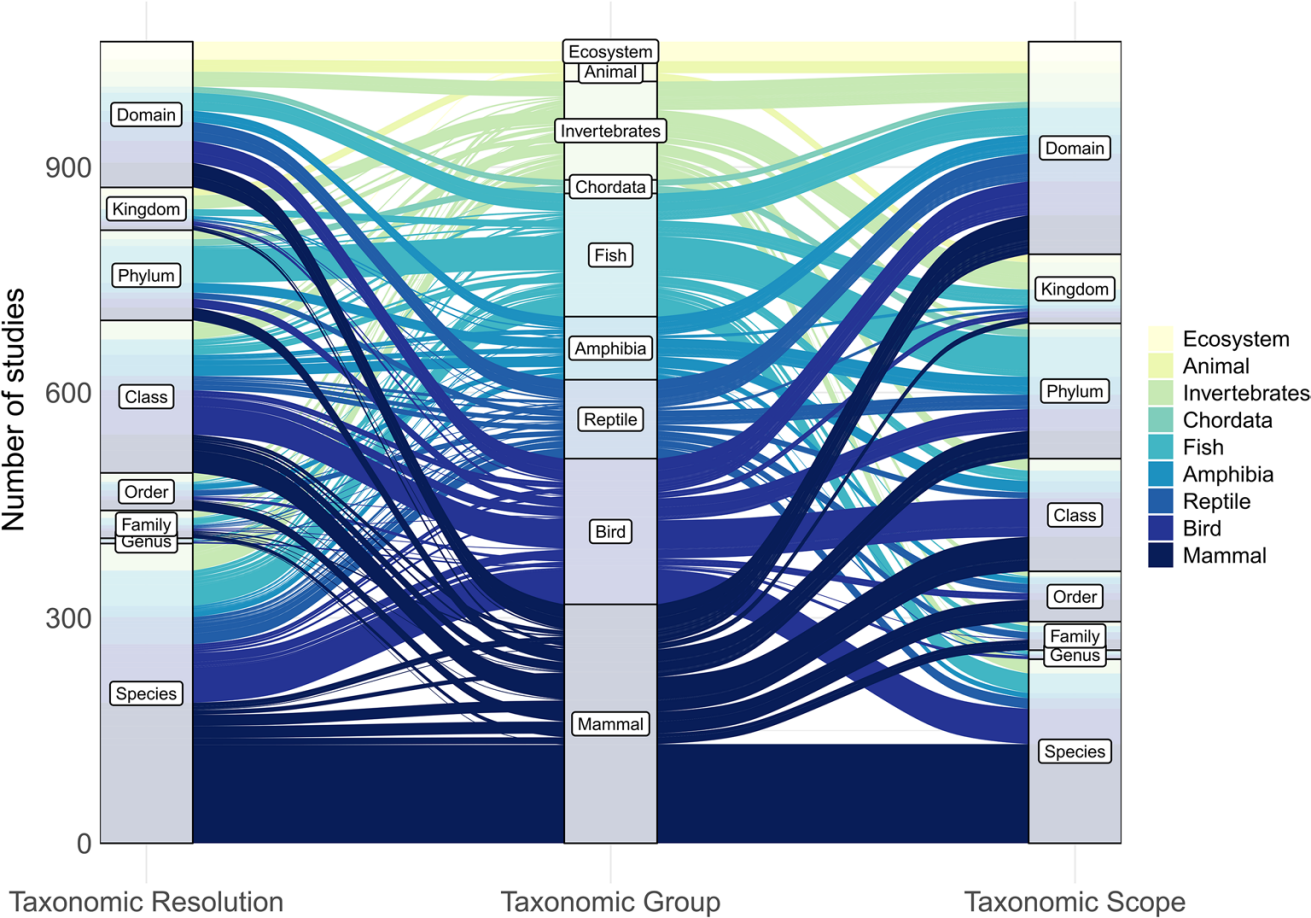
The difference in taxonomic resolution and taxonomic scope of retrieved threat mapping literature among animal taxonomic groups. Taxonomic resolution is the lowest taxonomic level that was mapped as an independent population unit, thus indicative of how taxonomically detailed the threat mapping application was. Whereas, taxonomic scope is the lowest taxonomic level that includes all species for which threats were mapped within the article. The width of the flows represents the number of articles

**Fig. 8:**
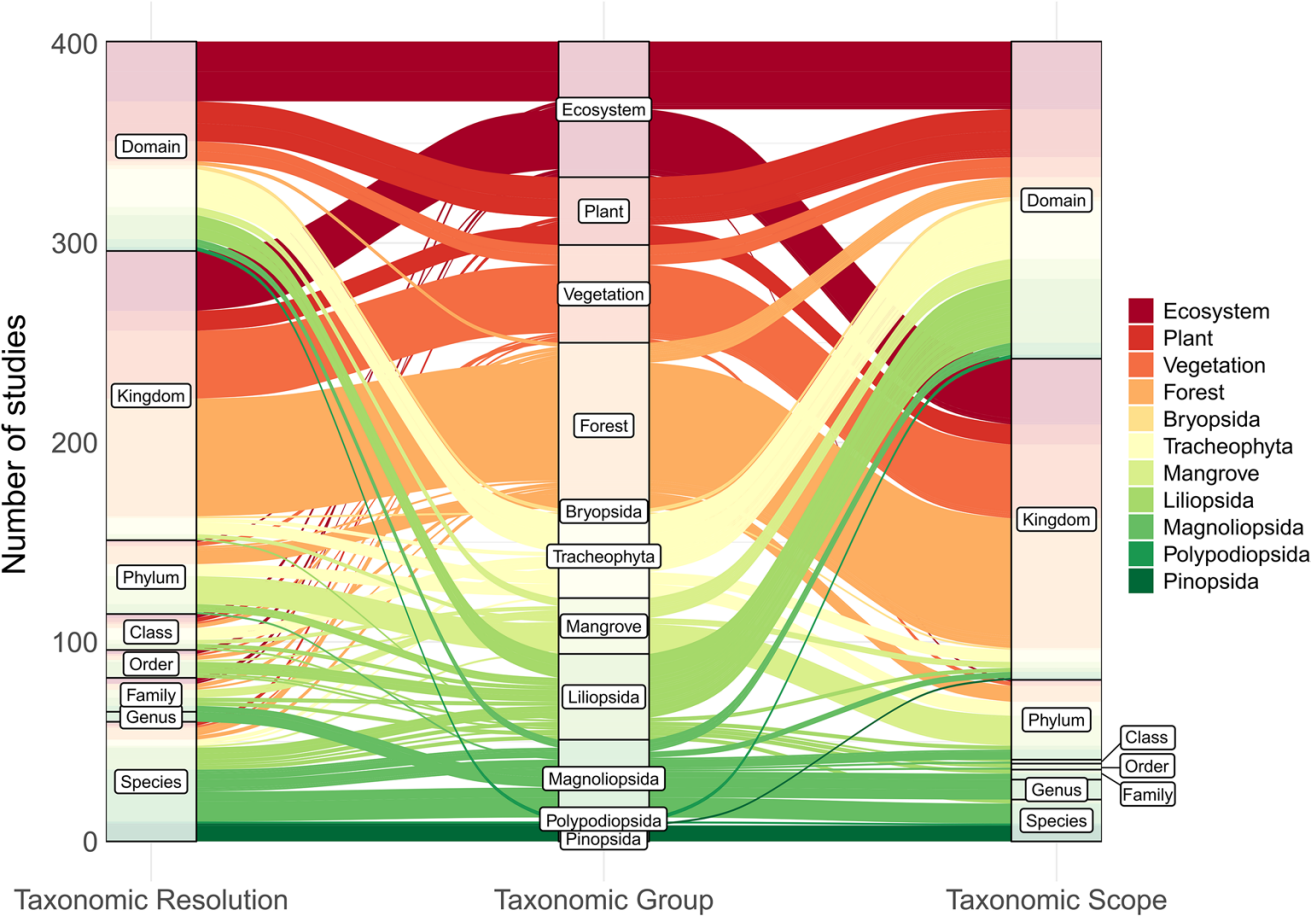
The difference in taxonomic resolution and taxonomic scope of retrieved threat mapping literature among plant taxonomic groups. Taxonomic resolution is the lowest taxonomic level that was mapped as an independent population unit, thus indicative of how taxonomically detailed the threat mapping application was. Whereas, taxonomic scope is the lowest taxonomic level that includes all species for which threats were mapped within the article. The width of the flows represents the number of articles

In addition to differences in overall research effort between kingdoms, consideration of taxonomic resolution and taxonomic scope revealed differences in the ways that each kingdom was studied. For example, 79% of animal articles mapped species at class-level or below and 49% of contained species-specific threat maps (Fig. 7). By comparison, 28% of plant articles mapped threats at class-level or below with 16% that mapped threats at a species-specific level (Fig. 8). Conversely, threats to plants were more likely to be mapped at a kingdom or domain level (43% and 19% of plant articles respectively) than threats to animals (4% and 7% of animal articles respectively, Fig. 8). Furthermore, there was a greater proportion of single-species articles among animal threat mapping articles (34%, *n* = 245) than among plant threat mapping articles (8%, *n* = 21, Fig. 7). Therefore the evidence on the spatial occurrence of threats is both more numerous and more taxonomically specific for animals than for plants.

#### Distribution among threats

Articles were collected that represented all 41 relevant categories of threatening human activities and direct human-initiated processes, in addition to six ‘other or unspecified’ categories. All articles were classified as examining at least one threat.

**Fig. 9:**
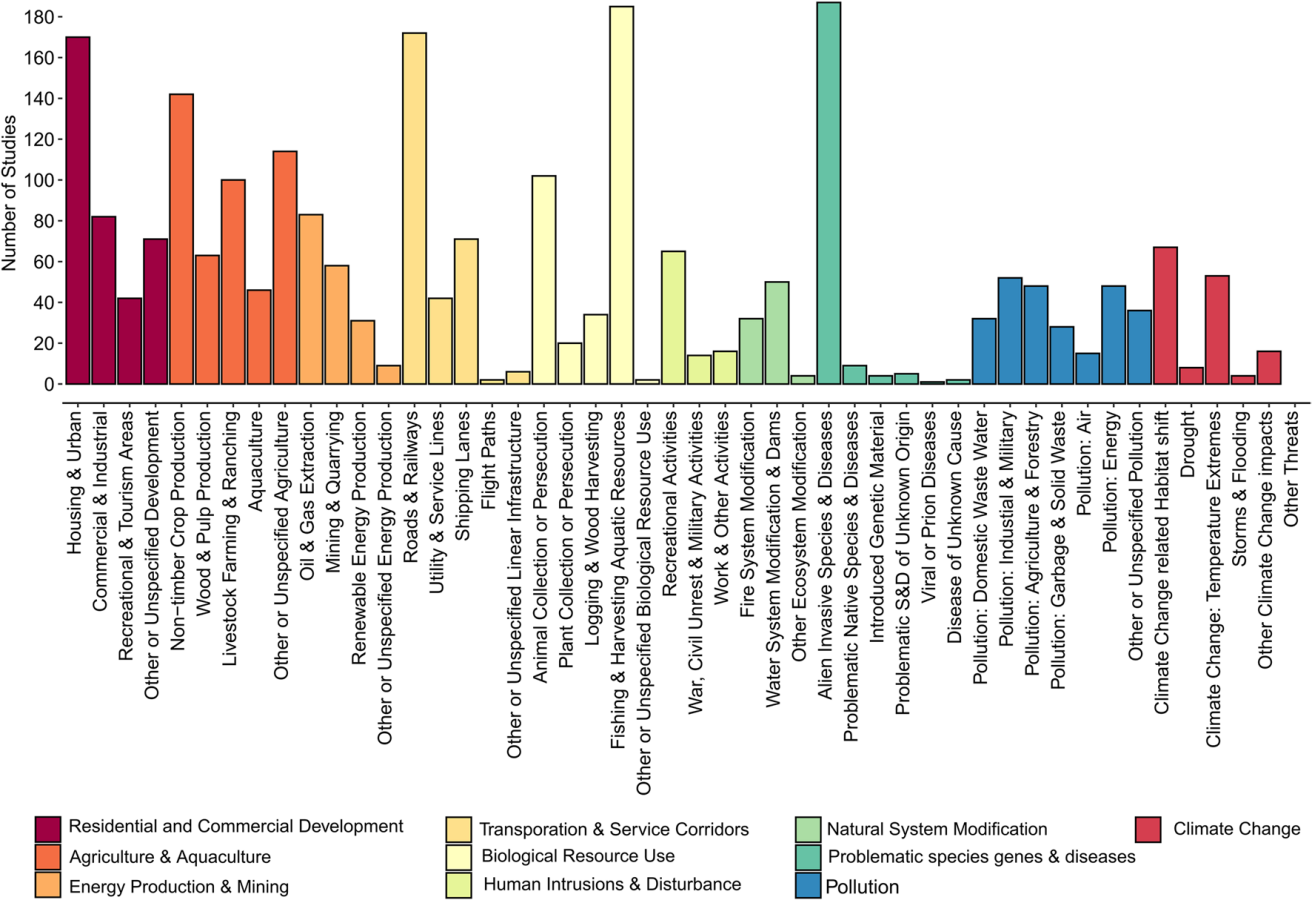
The number of threat mapping articles that mapped each threat. Threats were classified according to the IUCN threat classification scheme [38]. Colours indicate groups of thematically similar threats (i.e. level 1 in the threat classification scheme)

Eight threats occurred in 100 or more articles. These were: alien invasive species or diseases (187 articles), fishing and other aquatic resource harvesting (184), roads and railways (172), residential and urban development (170), non-timber crop agriculture (142), unspecified agriculture (114), hunting and collection of terrestrial animals (102), and livestock farming (100, Fig. 9). Excluding ‘other or unspecified’ categories, seven threats were mapped in less than ten articles. These were: problematic native species (nine articles), climate-change-induced drought (eight articles), problematic species of unknown origin (five articles), climate-change-induced storms or flooding (four articles), introduced genetic material (four articles), flight paths (two articles), and viral or prion-induced diseases (one study, Fig. 9).

When threats were grouped thematically, ‘Agriculture and aquaculture’ was overall the most frequently mapped threat group (323 articles), followed by ‘Biological resource exploitation’ (314 articles), ‘Residential and commercial development’ (280 articles), and ‘Transport and utility lines’ (268 articles, Fig. 9). ‘Problematic species’, ‘Pollution’, ‘Energy and mining’, and ‘Climate change’ each occupied between 101 and 204 articles. Meanwhile, two threat groups featured in less than 100 articles, which were: ‘Human intrusion and disturbance’ (87) and ‘Natural system modification’ (81, Fig. 9).

##### Terrestrial

**Fig. 10:**
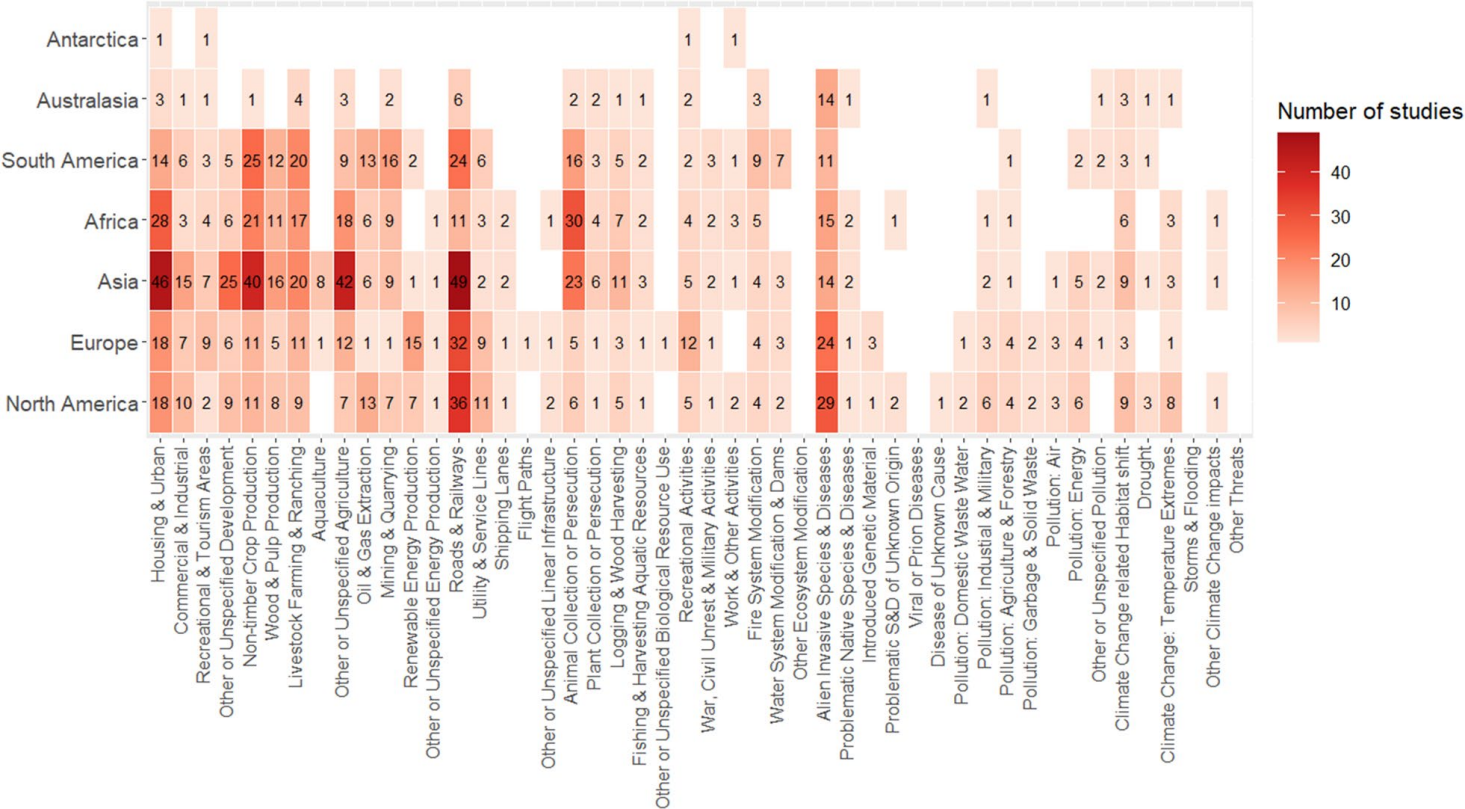
The representation of different threats in the terrestrial threat mapping literature and differences among geographic regions

The distribution of research effort among threats differed somewhat with geographic location, though three threats (alien invasive species, roads and railways, and residential development) were widely studied across the terrestrial realm (Fig. 10). Roads and railways occupied 10–29% of articles on the Asian, Australasian, European, North American, and South American continents. Meanwhile, Alien invasive species occupied 50% of articles in Australasia, 23% of articles in North America, 21% of articles in Europe, and 8–14% of articles in Asia, South America, and Africa. Furthermore, Residential development varied widely, from 11% in Australia to 27% in Africa (Fig. 10).

When threat groups were considered collectively, agricultural threats were also widespread, although of greater importance in the global south. Articles mapping agricultural threats for Asia, Africa, and South America, collectively occupied 46%, 51%, and 42% of all terrestrial threat maps on these continents respectively. This was much larger than in Europe, North America, and Australia for which agricultural threats represented 20–25% of the total threat mapping articles on each continent.

There were some differences in terrestrial threats among continents. Europe contained a relatively high proportion of articles mapping recreational disturbance (10%) and renewable energy production (12%) in comparison to the other continents (Fig. 10). Whereas, animal resource exploitation was mapped at an above average rate for Africa, South America, and Asia (28%, 18%, and 13% respectively, Fig. 10). Four threats were mapped in the single study of terrestrial Antarctica, these were residential development, recreational development, recreational disturbance, and work and other disturbance.

##### Freshwater

**Fig. 11:**
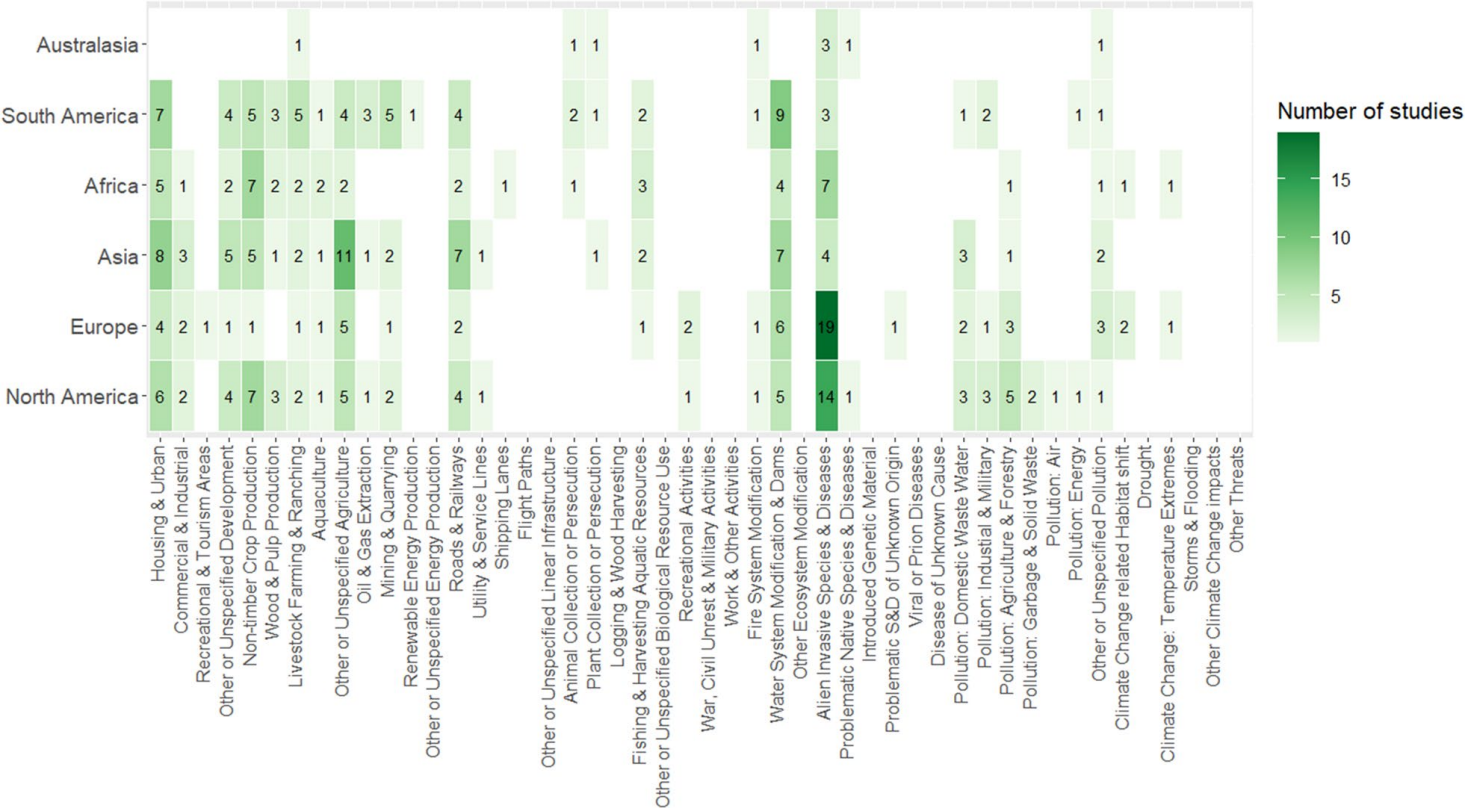
The representation of different threats in the freshwater threat mapping literature and differences among geographic regions

32 threats were mapped in the freshwater realm, eleven of which were mapped on two continents or fewer (Fig. 11). Alien invasive species was the most ubiquitously mapped freshwater threat, being the most heavily mapped on Australasia (75% of articles), Europe (51%), North America (39%) and Africa (32%, Fig. 11). Alien invasive species was also the only freshwater threat for which threat mapping articles were found on all continents. ‘Water system modification and damming’ was also reasonably widespread, ranging from 14 to 31% of threat mapping articles on each continent and was mapped on every continent apart from Australasia (Fig. 11).

##### Marine

**Fig. 12:**
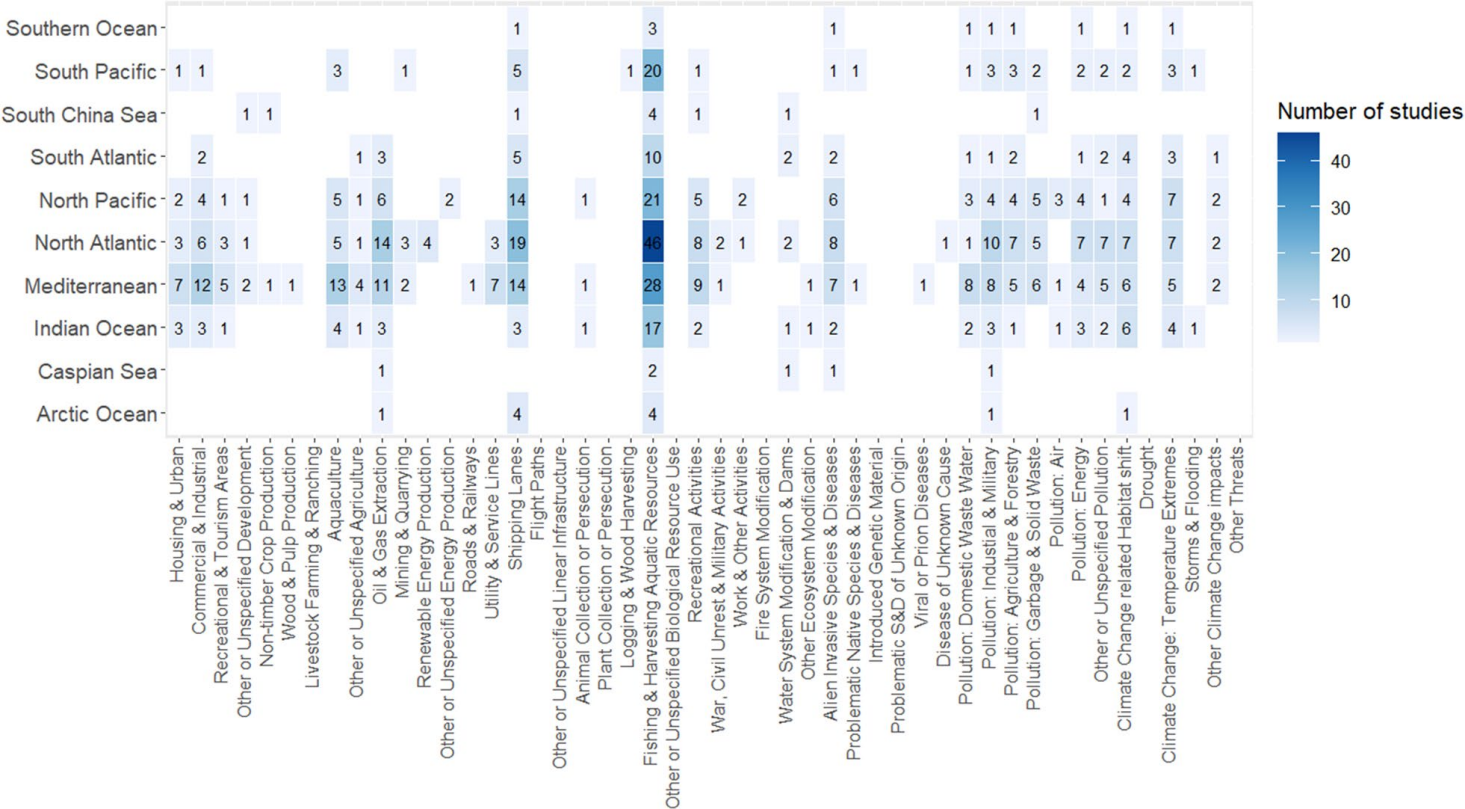
The representation of different threats in the marine threat mapping literature and differences among geographic regions

Threat mapping articles were found for 38 of 47 threats in the marine realm, with 13 threats mapped on two continents or fewer. Fishing and aquatic resource harvesting was mapped across all marine regions, ranging from 50 to 100% of articles on each marine region (Fig. 12). Study of shipping lanes was similarly widespread; only absent from the Caspian Sea. However, the contribution to the overall body of threat mapping literature found in each region was lower (12–44%, Fig. 12). Oil and Gas extraction, aquaculture, and commercial development were also studied with above average frequency across most marine regions (Fig. 12). Furthermore, some form of pollution was mapped for every marine region and, when considered collectively, represented 11–47% of articles on each marine region (Fig. 12).

#### The number of threats considered in each study

**Fig. 13:**
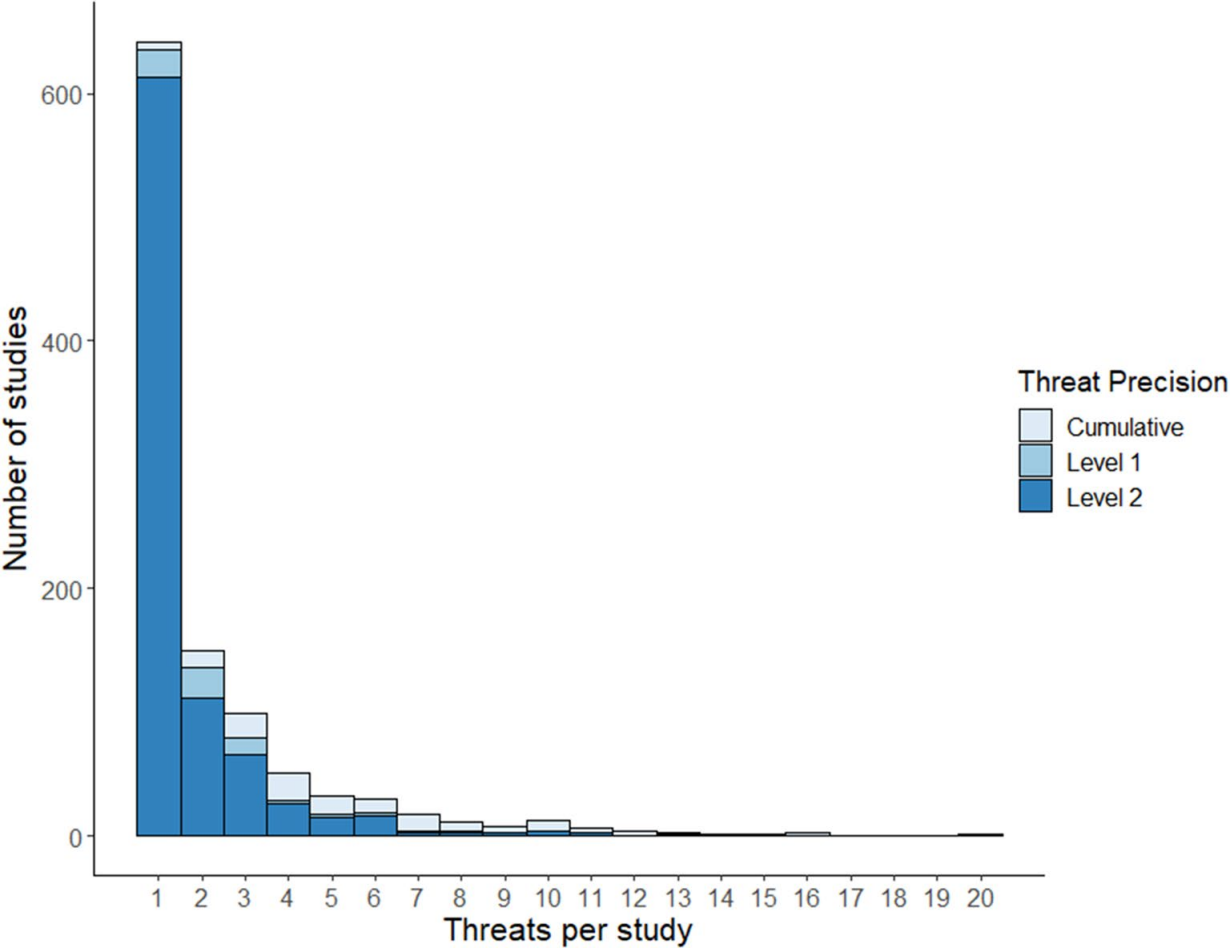
The number of different threats mapped within each threat mapping study, indicating the thematic precision at which threats were mapped. ‘level 2’ is the finest level of precision (e.g. Oil and Gas Drilling), ‘level 1’ (e.g. Energy production and mining) is less precise, and ‘cumulative’ indicates where multiple thematically different threats were mapped as one

Threats tended to be mapped in isolation. 60% of threat mapping articles mapped a single threat, whilst 91% of articles mapped five or fewer (Fig. 13). Most threats were mapped at the most precise level (862 at level 2, e.g. Residential an Urban Development), and fewer were mapped cumulatively (different threats combined into one index, 136 articles) or at level 1 (71, e.g. Residential and Commercial Development). Threat precision decreased as the number of threats per study increased (Fig. 13). For example, 96% of articles that mapped one threat did so at level 2 precision. Whereas, 74% of articles mapping ten or more threats and all articles mapping 14 or more threats, did so cumulatively (Fig. 13). Therefore, most evidence is contained within single-threat articles and thematic precision tended to be lost as the number of threats increased.

### Mapping the quality of articles relevant to the question

#### The methods used to map the spatial occurrence of threats to species

A range of methods were employed to map the threats to species, with many articles using more than one method. 23% of articles used multiple methods to map threats and 16% used multiple methods to map the population. Overall, ground-level survey methods were the most frequently-used source of data used to map the population (used in 32% of articles), followed by existing databases (24%), remote sensing (16%), and sourcing from the existing literature (15%, Table 5). Meanwhile, existing databases were the most frequent source of data on threats (used in 31% of articles), followed by ground-level survey methods (27%), remote sensing (22%), and sourcing from the existing literature (15%, Table 5). Far fewer articles utilised expert elicitation methods to map the population (5%) or threat (7%). Furthermore, modelling techniques were more commonly utilised to map the threat (21%) than the population (11%, Table 5). Finally, a small number of articles were found that used a pre-calculated metric of threats (2%) in comparison to 22% of articles that used a protected area or pre-calculated high biodiversity area to map the population (Table 5).

**Table 5:** The methods used to map the spatial occurrence of threats to species

Type	Method	Number of Articles using each method/data source for each question component	
		Population	Outcome (Threat)
Primary Collection	Survey	346	292
	Expert Elicitation	49	75
	Remote Sensing	172	237
Secondary Collection	Database	261	330
	Literature	164	160
Primary Synthesis	Modelled	119	221
Secondary Synthesis	Pre-calculated Metric (or PCHBA)	77	17
	Protected Area	160	–
	Other	5	9

PCHBA, pre-calculated high biodiversity area (e.g. Biodiversity Hotspots [20] or Key Biodiversity Areas [111])

Some of the methods used to map the spatial occurrence of threats to species did not fit within the classification used. Nine articles used other methods to map threats and five used other methods to map the population (eleven collectively). Of these, five used social media and other online platforms [100–104], three used museum or other archives [105–107], and for three articles it was unclear how the locations of the mapped threats were determined ([108–110], Table 6).

**Table 6:** Descriptions of the methods used in the eleven articles where the method of mapping either the population or outcome component could not be classified under the framework used

Study	Other method		Description
	Population	Outcome	
Abreo 2019 [100]	X	X	Social Media
Hausmann et al. 2019 [101]		X	Social Media
Jensen et al. 2019 [102]	X	X	Social Media and online trading platforms
Kitzes and Shirley 2016 [108]		X	Unclear how the locations of the dams were determined
Kraus et al. 2012 [105]		X	Museum specimens (in combination with other methods)
Lin et al. 2019 [104]		X	Social Media
Montevecchi et al. 2012 [109]		X	Unclear how the locations of the oil slick or the extent of oil coverage were determined
Sonricker et al. 2012 [103]	X	X	Social Media and online news
Tancell et al. 2016 [110]		X	Unclear how the locations of different marine resource-use designations were determined
De Castro et al. 2017 [106]	X		Museum records (in combination with other methods)
Lawler et al. 2003 [107]	X		Natural Heritage records (in combination with other methods)

#### Datasets used to map threats to species

Across the 408 articles that used data from existing databases to map either the population or threats, 142 population databases and 291 threat databases were used. Databases were recorded as reported in the articles, therefore some of the underlying datasets may overlap (e.g. IUCN Red List and BirdLife). IUCN Red List and government sources of data were the most common sources for both population and threat data (Table 7). Furthermore, the source population database was not identified in 57 articles and the source threat database was not identified in 32 articles (Table 7).

**Table 7:** Databases recorded from articles where the population data were gathered from an existing database and the threat data used were either a pre-calculated metric of threat or gathered from an existing database

Population data sources	Number of articles	Threat data sources	Number of articles
IUCN	53	Government Data	45
Government Data	17	IUCN	15
BirdLife	12	FAO	10
GBIF	6	Land Cover/Land Use	10
FishBase	5	Human Footprint	9
Nature Serve	5	Marine Impact (Halpern)	8
Aquamaps	2	WorldClim	7
Biodiversity Hotspots	2	GBIF	5
NOAA	2	Climate Research Unit	3
Other	125	Other	246
Source not found	57	Source not found	32

‘Source not found’ indicates that although the paper used existing data, neither reviewer found information on the source of the data

### Limitations of the map

#### Search strategy

The search strategy included two databases and 13 organisational websites specifically designed to target grey literature, yet journal articles were the primary document type among articles included in the review. Given this systematic map was global in scope, we chose to search the websites of organisations that operate internationally. It is possible that smaller-scale organisations involved with conservation planning and management could contain additional applications of threat mapping. Contacting the organisations directly may have also been a more successful method of identifying novel unpublished research applications than online repositories. Therefore, an organisational website search targeting smaller organisations directly could result in further applications of threat mapping being found.

As previously identified above, language also represents a limitation of the search strategy. The contextual and linguistic nuances associated with this investigation presented significant challenges and thus reinforced the decision only to include articles published in English. However, we recognise that this presents a risk of overlooking articles from non-English speaking countries. Therefore, we openly invite researchers with sufficient language resources to replicate this analysis for threat mapping research published in non-English languages. Regardless, it should be acknowledged that the English-only search and imperfect ability to target organisational resources could have contributed to the uneven distribution of literature observed.

#### Eligibility criteria

The multi-faceted and diverse nature of the retrieved literature meant that a large number of articles conceptually challenged the eligibility criteria. For example, NDVI was not considered a valid proxy for wild species presence as it alone cannot distinguish between natural and human vegetation [112]. However, NDVI was considered a valid method for measuring the extent of threats such as logging where other evidence was provided to confirm the eligibility of the population and threat. Nonetheless, levels of consistency still met the proposed threshold for the abstract and full-text screening stage. Challenging articles were discussed when the review team met to consolidate disagreements and efforts were made to update the coding tool. However these nuances were not always generalisable to changes in the eligibility criteria. Therefore, a list was kept of recurring examples of such articles and how they related to the eligibility criteria (Additional file 3).

This systematic map was limited to articles that presented visually the geographic distribution of threats, therefore it does not represent an exhaustive collection of all the articles on in-situ threats that exist. For example, fifty percent of articles excluded at the full-text screening stage studied an otherwise relevant population and threat but either did not study it spatially or did not visually present the geographic distribution of these two components. Therefore, more evidence on the spatial distribution of threats to species could be generated by gleaning the country or study-site location from any study investigating the effect of a relevant threat on populations of animals or plants.

Furthermore, consistency among reviewers at the title-screening stage was slightly lower than desired. All reasonable efforts were made to minimise the impact of this on the review findings within the time available, such as reviewing all disagreements again at abstract level. However, it is possible that some relevant articles were excluded at this stage.

## Conclusions

This systematic map collected and consolidated literature that mapped the threats to animal or plant species across the world. The number of threat mapping articles has increased through time from five articles published in 2000 to a maximum of 149 in 2018. The final database consists of 1069 articles, covering all 41 relevant IUCN threat categories and an additional six ‘other or unspecified’ categories. Of these articles, most studied a single threat. Therefore, the systematic map cannot necessarily provide information on the relative impact of particular threats on species in selected areas, but it can be used as a starting point for detailed syntheses of the available evidence and an organised repository of relevant information for use in threat reduction and spatial planning for conservation.

Evidence was distributed unevenly among every study attribute we assessed: spatial scales, geographic locations, ecological realms, taxonomies, and threats. The freshwater realm was the subject of substantially less research effort than the terrestrial and marine realms. Global threat maps were disproportionately numerous in all ecological realms, while the more focussed articles tended to be conducted at a sub-national or local scale in the land or sea territories of developed nations in the global north (particularly the USA). In terms of target species, animals, particularly mammals, birds, and fish, received greater research attention in comparison to other taxonomic groups, with non-vascular plants, reptiles, and amphibians receiving less attention. Similarly, some threats were more frequently studied (alien invasive species, harvesting aquatic resources, roads and railways, and residential development), whilst, other threats were seldom studied (non-alien invasive species, genes or diseases, climate-change-induced drought, storms or flooding, and flight paths).

### Implications for policy/management

#### Implications for the Post-2020 Global Biodiversity Framework

This systematic map found generous amounts of threat mapping evidence of direct relevance to the post-2020 Global Biodiversity Framework [113, 114]. In particular, maps of agriculture and aquaculture, alien invasive species, and residential development were in relatively high abundance. This evidence could be synthesised or used directly to inform biodiversity-inclusive spatial planning.

The high abundance of articles mapping the threat of roads and railways indicates that specific acknowledgement of this threat under the post-2020 framework might be beneficial to preventing species extinctions. The evidence for various impacts of roads on species is extensive [115–140], though efforts to reduce the direct threat of transport infrastructure are challenged by the critical role of transport in economic and social development [141–143]. Furthermore, a 60% increase in global road and rail network length is expected by 2050 [144]. Therefore, there is reason to consider the benefits to biodiversity of specifically targeting roads and railways under the post-2020 framework.

Biological resource use has been identified as the most pervasive threat to species [64] and is specifically featured in the Post-2020 framework. Threat mapping evidence found on it was heterogeneously distributed and clustered around terrestrial Asia, Africa, and South America, where there is higher reliance of communities on wild meats for sustenance, and presence of illicit wildlife trades [145–147]. Nonetheless, hunting and persecution of animals also occurs in Europe and North America [148–153] where mapping evidence for this threat was limited. Therefore, this systematic map will be useful for the implementation of targets on terrestrial biological resource use in Asia, Africa, and South America but more evidence is needed elsewhere. Furthermore, here biological resource use in the terrestrial and marine realms does not distinguish between intentional and unintentional effects (e.g. bycatch), which each require different methods of threat reduction [154–156]. Therefore, further targeted synthesis of articles that map the threat of biological resource exploitation could have large benefits for threat reduction activities on land and marine spatial planning.

#### Implications for national and local policy/management

Decision-makers at a local and national scale will be able to use the database of articles to quickly and easily identify evidence of relevance to their specific application. Further critical appraisal and extraction of the magnitude of threats for each study are necessary to translate the evidence into threat reduction activities. Therefore, the systematic map and corresponding database of articles presents a valuable starting point for evidence-based decision-making for threat reduction at local and national scales.

### Implications for research

This systematic map has reinforced the need to fill knowledge gaps in the previously identified areas of bias (taxonomy, geography, and ecological realm). In particular, plants and freshwater systems were starkly under-studied. Research effort in these areas was not only low overall, but low in particular foci expected to be of high conservation need. For example, articles of biological resource use on plants and in the freshwater realm were scarce, despite 42% of threatened plant species and 41% of threatened freshwater species (animals and plants) being threatened by biological resource use [157]. Our findings also agree with others that there are geographic biases towards North America and Europe and against Africa and areas of South America and Asia [49, 56, 158]. The above gaps are widely acknowledged but efforts to fill them continue to be hampered; we add our voices to those who call for more research into these underrepresented regions, countries, continents, taxa and ecological realms, which are equally threatened by human activities.

The disproportionately high number of global maps we found will feed into a wider debate on the purpose of global conservation priority mapping [159]. We found almost as many global-scale threat maps as threat maps of any other spatial scale for either Africa or South America. Global scale analyses overlook contextual specificities and complexities in decision-making that influence the success of conservation interventions [160–163]. Meanwhile, actions to implement global frameworks like the Post-2020 Global Biodiversity Framework occur at a national level, yet a scarcity of maps has been reported in national strategies for both climate change and biodiversity [164, 165]. Therefore, we encourage researchers and individuals responsible for conducting global assessments to explore synthesising the collective findings from smaller-scale threat maps before conducting a global-scale map, and to prioritise filling knowledge gaps at the national level, where implementation is most likely to occur.

Specific knowledge gaps were observed in land-based threats to marine systems and in the simultaneous study of multiple threats. There is an extensive body of literature to support the importance of land-based threats to the marine realm [166–172] and the importance of managing multiple threats [17, 71, 72, 173–176]. However, we found that most articles investigated single threats and few threat-mapping articles of the marine realm investigated land-based threats, representing critical gaps in knowledge. Therefore, specific research that maps the land-based threats to the marine realm, and the spatial interactions between different threats, could have large benefits for conservation outcomes.

This systematic map of the literature details the uneven distribution of retrieved threat mapping literature across threats, taxonomies, geographies, and methodologies. It highlights clusters in knowledge of relevance to the implementation of the Post-2020 Global Biodiversity Framework and draws attention to knowledge gaps that may distort our understanding of where and how human activities threaten species to inform future research. Moreover, the interactive database of threat mapping literature provides a user-friendly tool that makes this area of research more accessible to conservation policy-makers and practitioners.

## Supplementary Information

**Additional file 1. **Examples of threat maps: Example threat maps from some of the included threat mapping articles.

**Additional file 2. **Supplementary search details: Specifics of how each database and organisational websites were searched.

**Additional file 3. **Supplementary eligibility information: Further details of what priority areas were considered a valid proxy for species presence within this study and examples of articles that challenged the eligibility criteria and how they were treated.

**Additional file 4. **Searchable database of studies: A multiple page workbook containing the coding tool and data collected per article, bibliography, term definitions, disaggregated dataset, and article searching tool.

**Additional file 5. **Excluded full-texts: Bibliographic details of all studies that passed abstract screening but were not included in the final systematic map and the reasons why they were excluded.

**Additional file 6. **Geographic distribution tables: Details of the number of relevant threat mapping articles found for each land area and marine territory.

**Additional file 7.** Completed ROSES Form.

## Data Availability

The data generated and analysed in this systematic map is provided in the supplementary materials to this published article.
