# Supplementary material for: The scope and extent of literature that maps threats to species globally: a systematic map
Source: Environ Evid. 2022 Jul 9;11:26. doi: 10.1186/s13750-022-00279-7 (PMC11378821; doi:10.1186/s13750-022-00279-7)
Supplement: Supplementary file 1 — Additional file 1. Examples of threat maps: Example threat maps from some of the included threat mapping articles. [file 13750_2022_279_MOESM1_ESM.docx]

README

Additional File 1.doc: Examples of threat maps: Example threat maps from some of the included threat mapping articles.

Table 1. Visual examples of threat maps

| In-text Citation | Figure legend extract | Map |
| --- | --- | --- |
| Lieske *et al* 2020 [1] | “Fig. 5. Risks to “large gulls” from the following threats: (a) gillnet and (b) longline fisheries, (c) ship-sourced oil pollution, and (d) all threats…“Risk” is the product of threat intensity, relative [gull] abundance, and sensitivity” | 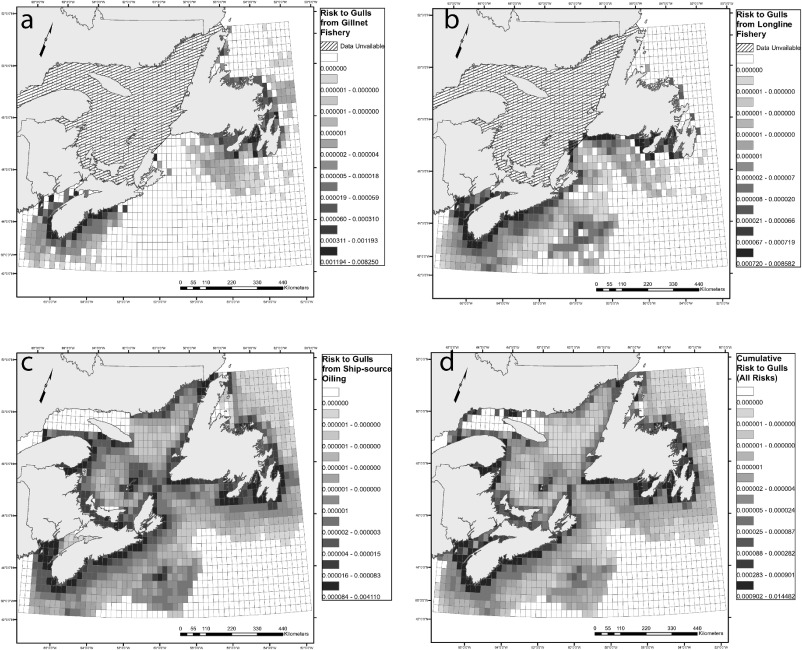 |
| Morelli *et al* 2020 [2] | “Fig. 3. A forecasting map of avian roadkill-risk in Europe, based on the combination between road density (total length in km) (above, left) and cumulative risk of roadkill in avian species assemblages (above, right) in each square of a grid of 50 × 50 km covering the whole European continent….” | 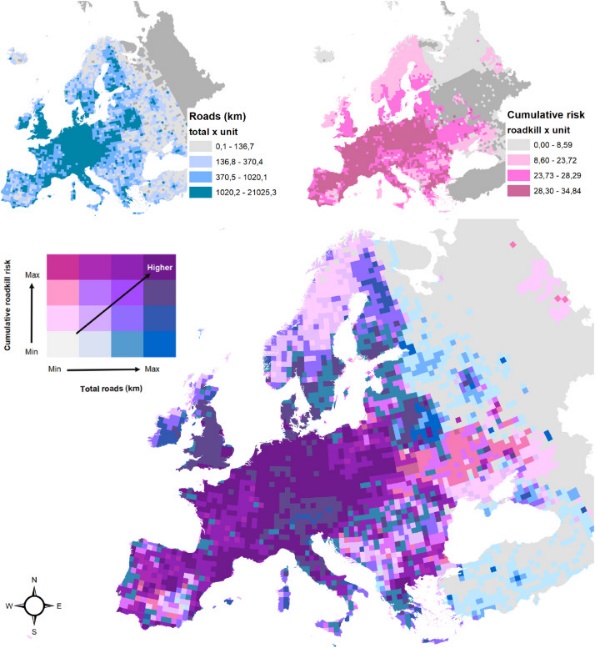 |
| Dutta et al 2017 [3] | (top) “Fig1. Maps show location of the study are in the eastern part of India and in the state of West Bengal and green patches are the protected forest (PF) boundary under Durgapur Forest Range and its association with settlement and road lines in black dots and red colours line respectively.”  (bottom) “Fig4. Forest fragmentation under protected forest area of Durgapur Forest Range in 1990 (A) and 2015 (B)” | 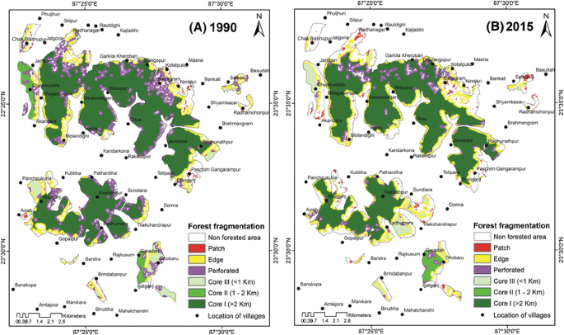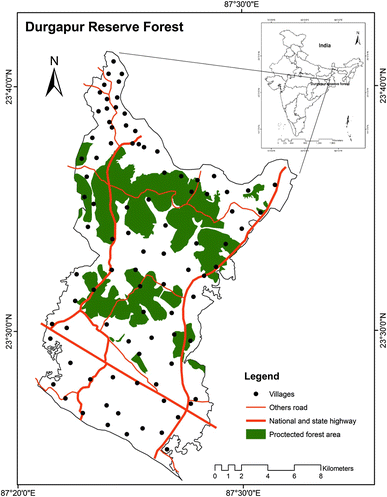 |
| Ortiz and Torres 2019 [4] | **“Figure 6.** Large provincial producers of high value crops of banana and pineapple in Mindanao. Points indicate threatened species occurrence (tree species and fauna), and circles show identified pineapple and banana plantations with a 10-km buffer.” | 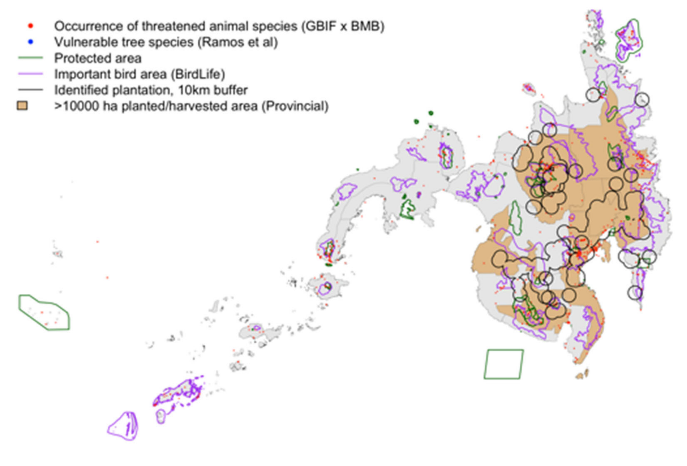 |
| Abade et al 2018 [5] | “Fig1 Spatial distribution of the camera-trap stations (red shaded circles) across the Ruaha landscape.”  Specifics of the human activities that occurred in each of the zones were provided in the text. | 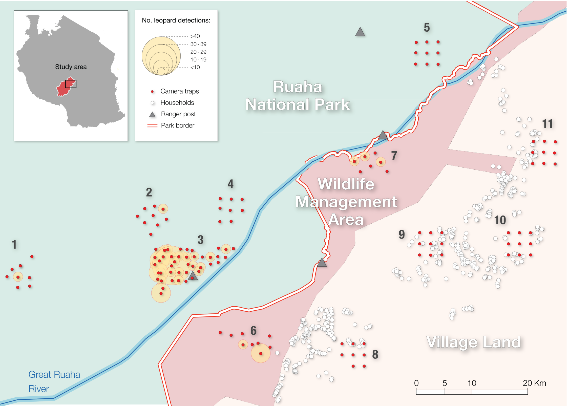 |
| Araújo and Wang 2015 [6] | “Fig. 2 (a) The distribution of Inia geoffrensis (question marks indicate unconfirmed occurrence) and (b) Sotalia fluviatilis and the locations of hydroelectric dams in central Brazil. The three watersheds (North Atlantic, Amazon and Araguaia–Tocantins) are indicated by dashed lines. The names of the numbered dams are provided in Table 2.” | 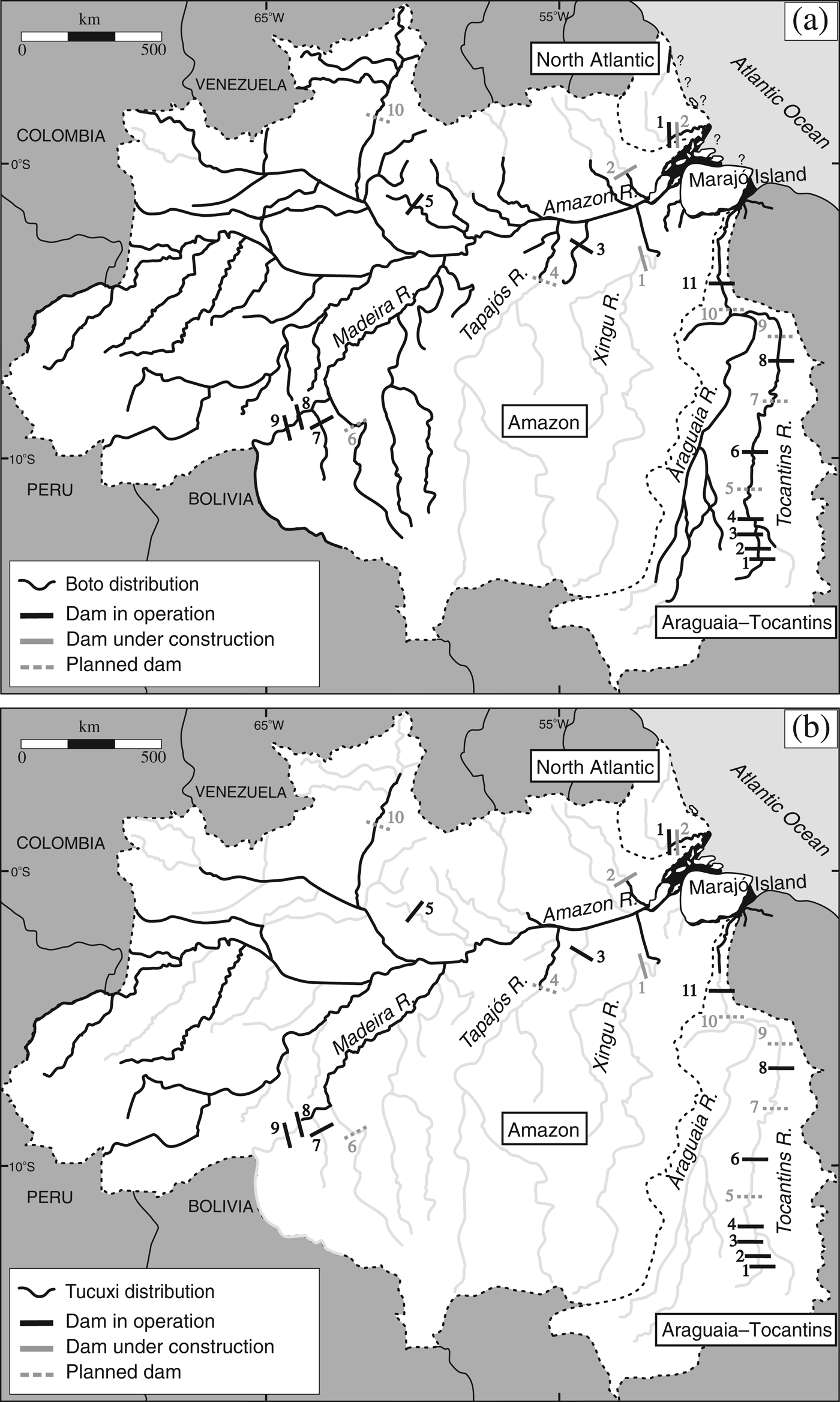 |

References

1. Lieske DJ, Tranquilla LM, Ronconi RA, Abbott S. “Seas of risk”: Assessing the threats to colonial-nesting seabirds in Eastern Canada. Marine Policy. 2020;115.

2. Morelli F, Benedetti Y, Delgado JD. A forecasting map of avian roadkill-risk in Europe: A tool to identify potential hotspots. Biological Conservation. 2020;249.

3. Dutta S, Sahana M, Guchhait SK. Assessing anthropogenic disturbance on forest health based on fragment grading in Durgapur Forest Range, West Bengal, India. Spatial Information Research. 2017;25:501-12.

4. Ortiz Andrea Monica D, Torres Justine Nicole V. Assessing the impacts of agriculture and its trade on Philippine biodiversity. (eds.). Cold Spring Harbor: Cold Spring Harbor Laboratory Press; 2019.

5. Abade L, Cusack J, Moll RJ, Strampelli P, Dickman AJ, Macdonald DW, et al. Spatial variation in leopard (Panthera pardus) site use across a gradient of anthropogenic pressure in Tanzania's Ruaha landscape. PLoS ONE. 2018;13.

6. Araújo CC, Wang JY. The dammed river dolphins of Brazil: Impacts and conservation. ORYX. 2015;49:17-24.
