# Supplementary material for: The scope and extent of literature that maps threats to species globally: a systematic map
Source: Environ Evid. 2022 Jul 9;11:26. doi: 10.1186/s13750-022-00279-7 (PMC11378821; doi:10.1186/s13750-022-00279-7)
Supplement: Supplementary file 2 — Additional file 2. Supplementary search details: Specifics of how each database and organisational websites were searched. [file 13750_2022_279_MOESM2_ESM.docx]

README

Additional File 2.doc: Supplementary search details: Specifics of how each database and organisational websites were searched

# 1. Specifics of the final search on each database

## 1.1 SCOPUS

SCOPUS claims to be an independent expert-curated abstract and citation database. The full source

title list of peer-reviewed journals covered by SCOPUS is accessible at:

<https://www.elsevier.com/solutions/scopus/how-scopus-works/content>

### In Command Line:

TITLE-ABS-KEY(( pressure OR threat OR risk OR stress OR footprint ) AND ( species OR

ecosystem OR wildlife OR fauna OR flora OR {spp.} OR {sp.} ) AND ( hotspot* OR map* OR

geographic* OR "gis" OR "spatial distribution" OR "spatial overlap" OR "spatial separation" OR

"spatial dynamics" OR "spatial variation" OR "spatial framework" OR "spatially explicit" OR

geospatial ) AND ( conservation OR biodiversity ))

AND PUBYEAR >1999 AND LANGUAGE(English)

### On page options:

None

## 1.2 Web of Science Core Collection

### Citation Indexes

- Science Citation Index Expanded (SCI-EXPANDED) --1970-present
- Social Sciences Citation Index (SSCI) --1970-present
- Arts & Humanities Citation Index (A&HCI) --1975-present
- Conference Proceedings Citation Index- Science (CPCI-S) --1990-present
- Conference Proceedings Citation Index- Social Science & Humanities (CPCI-SSH) --1990-present
- Emerging Sources Citation Index (ESCI) --2015-present

### In command Line:

TS =(( pressure OR threat OR risk OR stress OR footprint ) AND ( species OR ecosystem OR

wildlife OR fauna OR flora OR “spp.” OR “sp.” ) AND ( hotspot* OR map* OR geographic* OR

"gis" OR "spatial distribution" OR "spatial overlap" OR "spatial separation" OR "spatial dynamics"

OR "spatial variation" OR "spatial framework" OR "spatially explicit" OR geospatial ) AND (

conservation OR biodiversity ))

### On Page options:

Years: 2000 – 2020

Language: English

## 1.3 ProQuest Natural Science Collection

### Component databases

- Agricultural Science Collection (including AGRICOLA, 1970 – current)
- Environmental Science Collection (1970 – current)
- Biological Science database
- Biological Science Index (1946-current)
- ASFA: Aquatic Sciences and Fisheries Abstracts (1971-current)
- Earth, Atmospheric and Aquatic Science Database

### In command line:

Ti ( ( pressure OR threat OR risk OR stress OR footprint ) AND ( species OR ecosystem OR

wildlife OR fauna OR flora OR “spp.” OR “sp.” ) AND ( hotspot* OR map* OR geographic* OR

"gis" OR "spatial distribution" OR "spatial overlap" OR "spatial separation" OR "spatial dynamics"

OR "spatial variation" OR "spatial framework" OR "spatially explicit" OR geospatial ) AND (

conservation OR biodiversity ) )

OR Ab ( ( pressure OR threat OR risk OR stress OR footprint ) AND ( species OR ecosystem OR

wildlife OR fauna OR flora OR “spp.” OR “sp.” ) AND ( hotspot* OR map* OR geographic* OR

"gis" OR "spatial distribution" OR "spatial overlap" OR "spatial separation" OR "spatial dynamics"

OR "spatial variation" OR "spatial framework" OR "spatially explicit" OR geospatial ) AND (

conservation OR biodiversity ) )

OR If ( ( pressure OR threat OR risk OR stress OR footprint ) AND ( species OR ecosystem OR

wildlife OR fauna OR flora OR “spp.” OR “sp.” ) AND ( hotspot* OR map* OR geographic* OR

"gis" OR "spatial distribution" OR "spatial overlap" OR "spatial separation" OR "spatial dynamics"

OR "spatial variation" OR "spatial framework" OR "spatially explicit" OR geospatial ) AND (

conservation OR biodiversity ) )

### On page options (peer-reviewed):

- Peer-reviewed option selected
- Specific Year Range: 2000 – 2020
- Language: English

### On page options (grey literature):

- Peer- reviewed option unselected
- Specific Year Range: 2000 – 2020
- Language: English
- Publication types: Working Papers, Dissertations and Theses, Reports, Government and Official publications

## 1.4 Google Scholar

### In command line:

Allintitle: ( pressure OR threat OR footprint ) ( species OR ecosystem OR wildlife)

### On page options:

- Custom range: 2000 – 2020
- Sort by relevance (first 500 results collected)

# Results of organisational website searches

| Organisation | URL | Date Searched | Terms Used | Total Results | Number Screened | Number Relevant | Comments |
| --- | --- | --- | --- | --- | --- | --- | --- |
| WWF | <https://www.worldwildlife.org/> | 19.04.21 | threat report | ~ 2250 | 150 | 15 | First 15 pages screened (150 articles) in order of relevance |
| UNEP-WCMC | <https://www.unep-wcmc.org/resources-and-data> | 19.04.21 | threats | 21 | 21 | 2 |  |
| CBD | <https://www.cbd.int/kb/> | 22.4.21 | threats | 25 | 25 | 0 | Additional filters: excluded 'News & Announcements', 'Events', and 'Contacts' |
| IUCN | <https://www.iucn.org/resources/publications> | 22.4.21 | In title: 'threats' | 6 | 6 | 0 | Year greater than 2000 |
| IPBES | <https://ipbes.net/library> | 22.4.21 | no keyword used | 172 | 172 | 0 | documents searched: IPBES Journal publications, assessment reports and outputs. |
| RSPB | <https://www.rspb.org.uk/> | 22.4.21 | threats | 55 | 55 | 0 | Excluded document types =Wildlife pages, activities, appeals, News, Press releases, and volunteer opportunities |
| Fauna and Flora Int | <https://www.fauna-flora.org/> | 22.4.21 | threats | 100 | 100 | 0 |  |
| The Nature Conservancy | <https://www.nature.org/en-us/what-we-do/our-insights/reports/> | 22.4.21 |  | 66 | 66 | 1 | All articles on published reports page screened. Unable to filter or search further |
| Conservation International | <https://www.conservation.org/> | 22.4.21 | threats | 161 | 140 | 0 |  |
| Birdlife | <http://www.birdlife.org/> | 27.4.21 | threats publication | ~ 7830 | 60 | 0 | ordered by relevance |
| Blue Ventures | <https://blueventures.org/impact/publications/> | 27.4.21 |  | 177 | 177 | 5 |  |
| The Audubon Society | [www.audubon.org](http://www.audubon.org/) | 27.4.21 | threats | 1195 | 48 | 0 | In order of relevance |
| SCB | [www.conbio.org](http://www.conbio.org/) | 27.4.21 | threats | 218 | 218 | 0 | All with potentially relevant titles were narrative |
