# Supplementary material for: The scope and extent of literature that maps threats to species globally: a systematic map
Source: Environ Evid. 2022 Jul 9;11:26. doi: 10.1186/s13750-022-00279-7 (PMC11378821; doi:10.1186/s13750-022-00279-7)
Supplement: Supplementary file 3 — Additional file 3. Supplementary eligibility information: Further details of what priority areas were considered a valid proxy for species presence within this study and examples of articles that challenged the eligibility criteria and how they were treated. [file 13750_2022_279_MOESM3_ESM.docx]

README: Additional File 3.doc: Supplementary eligibility information: Further details of what priority areas were considered a valid proxy for species presence within this study and examples of studies that challenged the eligibility criteria and how they were treated.

# Pre-defined priority areas as a proxy for species presence

- 1. Examples of protected areas and pre-defined conservation priority areas that are considered a valid proxy for species presence
- IUUCN protected area categories 1a, 1b, 2, and 4 [1]
- Key Biodiversity Areas [2] includes:
  - Important Bird and Biodiversity Areas
  - Alliance for Zero extinction sites
  - Hotspot Ecosystems (Critical Ecosystem Partnership)
- Biodiversity Hotspots [3]
- Ramsar sites [4]
- UNESCO Natural World Heritage Sites [5]
- Marine protected areas [6]
  1. Examples of protected areas and pre-defined priority areas that are not considered to be valid proxies for population presence
- IUCN Protected Area categories 3,5, and 6 [1]
- UNESCO Cultural World Heritage Sites [5]
- UNESCO Man and Biosphere reserves [7]

## References

1. Dudley N. Guidelines for applying protected area management categories: Iucn; 2008.

2. IUCN. A global standard for the identification of key biodiversity areas. Version 1, 2016-2048.

International Union for Conservation of Nature; 2016.

3. Myers N, Mittermeier RA, Mittermeier CG, Da Fonseca GA, Kent J. Biodiversity hotspots for

conservation priorities. Nature. 2000;403:853-858.

4. Ramsar. Ramsar information paper no. 5 the criteria for identifying wetlands of international

importance (2007) [Available from: https://www.ramsar.org/document/ramsar-informationpaper-

no-5-the-criteria-for-identifying-wetlands-of-international.

5. UNESCO. Operational guidelines for the implementation of the world heritage convention.

(2019) [Available from: https://whc.unesco.org/

6. Kelleher G. Guidelines for marine protected areas: IUCN, Gland, Switzerland and Cambridge,

UK; 1999.

7. UNESCO. World network of biosphere reserves 2019.

# Examples of studies and how they relate to the eligibility criteria

## 2.1 Population

### **Criteria 1A:** Excluded if no evidence was given for the presence or distribution of a relevant species within the study area

*NDVI*

EXCLUDED if: The only measure of biodiversity used is NDVI. This is because some human land-uses can have high reflectance e.g. crops at peak growing season, livestock pasture and plantations

INCLUDED if: NDVI is used to study human disturbance (e.g. selective logging) to a known natural area.

### **Criteria 1B:** Excluded if the evidence provided was predictively modelled without evidence for species presence at the study site e.g. habitat suitability for the purpose of reintroduction

*No ambiguities were reported in applying this criteria*

## 2.2 Outcome

### **Criteria 2A:** Excluded if no data on where species and human-driven pressures co-occur were presented or the ‘threat’ considered did not fit the definition used here.

#### Not threatening

EXCLUDED if - the effect of the human-driven activity on the population is positive or the lack of evidence for a negative interaction is acknowledged by the authors. E.g adaptation of species to human-modified landscapes (e.g. urbanisation), the biodiversity value of different human land-uses.

INCLUDED if – a negative effect on species of the threat studied is demonstrated, justified in writing, or inherent (e.g. due to risk of direct mortality or injury).

#### Human-Wildlife conflict

EXCLUDED if - the subject of the impact is human or livestock rather than the wild species (e.g wildlife as reservoirs for human or domestic animal diseases, Wildlife attacks on humans or livestock, crop raiding).

INCLUDED if – The subject of the study is the human response to the above human-wildlife conflicts that results in a direct threat to wildlife (e.g. Raptor poisoning, pesticide exposure, retaliatory/revenge killings of wildlife, culling).

#### Active management

EXCLUDED if: The impact of livestock grazing on vegetation is considered in terms of the maintenance and active management of pasture (e.g. testing the intermediate disturbance hypothesis)

INCLUDE if: a study has mapped the occurrence of over-grazing of wild grasses

EXCLUDED if: The effectiveness of a culling regime is studied where the focus is on the management of a population rather than culling as a threat to wild species.

#### Proximity to human features

INCLUDED if: Proximity to human features is used as a proxy for a specific human activity (e.g. poaching) where further evidence is given to support the presence of that activity in the study site.

EXCLUDED if: The study investigates habitat selection of a particular species where a proximity to human infrastructure variable (e.g. roads or settlements) is among those included, without providing evidence or justification for what threat the proximity represents.

INCLUDED if: Proximity is used to estimate the threat of a human feature due to habitat fragmentation

### **Criteria 2B**: Excluded if the threat studied was relevant and studied in-situ but the occurrence was not mapped onto a geographic distribution.

#### Mapping study/sampling sites only

EXCLUDED if: Study/sampling sites were the only feature that was mapped and the results of the study showed that either species or threat presence did vary within the study site but this variation in presence was not mapped.

INCLUDED if: Study/sampling sites were mapped and the results showed that species and threat were present in some magnitude across all sampling points.

### **Criteria 2C:** Excluded if the threat considered was not human-driven.

*Wildlife pathogens, parasites or diseases (PPDs)*

INCLUDE if: the study considers the existing distribution of a PPD in a wild population that has arisen due to human activities and threatens the survival of that species. E.g studies of non-native PPDs, domestic to wildlife transmission, observed increased impact of PPD due to climate change, PPDs that have recently or suddenly emerged such as sudden oak death, chytrid fungus, or white nose syndrome.

EXCLUDE if: no evidence is presented to support the assumption of human influence on PPD presence, abundance or host susceptibility. For example, a disease with an acknowledged long history in the area such as anthrax.

#### Nitrogen deposition

EXCLUDE if: The variation in nitrogen content of soils/water is measured without distinguishing the anthropogenic contribution

INCLUDE if - The anthropogenic contribution is justified and evidenced

## 2.3 Study Type

### **Criteria 3A:** Excluded if the article was a narrative review in which no new synthesis of data was presented.

#### Systematic Reviews and meta-analyses

INCLUDE if: A study has systematically collected literature that individually meets the criteria used here and synthesised the collective findings.

INCLUDE if: The study mapped the geographic locations of field studies that found a negative impact of a human-driven pressure on species. The literature in this case is the metric that the study has used to map the threat distribution

EXCLUDE if: the context of the review is relevant but the locations of studies observing a negative impact of human-driven pressures on wild species have not been mapped or cannot be distinguished from those observing a positive or no effect

EXCLUDE if: The review is narrative with no novel synthesis of data

### **Criteria 3B:** Excluded if the presented distribution represented a historical, future, or potential distribution of threat to species.

#### Invasive alien species (IAS)

INCLUDE if: overlap between IAS and population is observed and full potential distribution of IAS within the study area is modelled

EXCLUDE if: overlap between IAS and population is predicted without any current/recent observations in those locations. For example if global spread of IAS is predicted in space or time based on analogous climate, transport pathways or other methods.

#### Climate Change

EXCLUDE if: study presents predicted future effects to species due to climate change e.g. predicted range restrictions, predicted sea level rise, invasive species spread, disease etc …

INCLUDE if: study presents where species are currently threatened by climate change e.g. where anthropogenic climatic changes have been observed, or where species are existing within narrow climatic niches, or at the extremes of their current climatic niche.

#### Could become threatening

EXCLUDE if: The population and threat are observed to overlap and the author(s) posit that the relationship could become negative in the future even though it is observed not to be at present (e.g. alien species). This is classed as a potential/future threat.

#### Development

EXCLUDE if: A development pressure has not yet been built. E.g. Identifying overlap between avian flyways and sites suitable for wind energy prior to development.

#### Human-driven catastrophes

EXCLUDE if: The risk of a potential catastrophe is studied (e.g. identifying potential sites at risk of oil spill)

INCLUDE if: The study maps the co-occurrence of species and observed human-driven catastrophes (e.g. Oil spills)

INCLUDE if: The study maps the existing threat to wild species from the infrastructure and activities of potential catastrophe-causing industries (e.g. Oil exploration)

### **Criteria 3C:** Excluded if the spatial context could not be determined (e.g. due to insufficient reporting), or the threat was not studied in-situ (e.g. theoretical, lab-based, or experimentally applied).

#### Simulations

EXCLUDE if: a future or theoretical distribution is simulated

INCLUDE if: simulation methods are used to model a current threat distribution for which the input included observed data on species presence and human-driven pressure (e.g illegal activities).

#### Experimental designs

EXCLUDE if: the study inflicts pressure in order to study a response within populations or individuals rather than observing existing human activities. For example, a study that studied the physiological response of fish to recreational angling by catching fish.

INCLUDE if: techniques such as exclusion are used to test the effect of a threat on a species in a location where it already occurs.

#### Expert elicitation

INCLUDE if: Surveys using interviews and/or questionnaires of indigenous communities, or academic experts are used to map the threat distribution. It is a commonly used method to study the extraction of bushmeat, non-timber forest products, and medicinal plants.

EXCLUDE if: The results of the survey do not generate information on where geographically the studied activities occurred. For example, if distances travelled to extract resources or offtake were reported without mapping the location of settlement.
